# Stabilization of *Bacillus subtilis* Spx under cell wall stress requires the anti-adaptor protein YirB

**DOI:** 10.1371/journal.pgen.1007531

**Published:** 2018-07-12

**Authors:** Daniel F. Rojas-Tapias, John D. Helmann

**Affiliations:** Department of Microbiology, Cornell University, Ithaca, NY, United States of America; Indiana University, UNITED STATES

## Abstract

Spx is a global transcriptional regulator present in low-GC Gram-positive bacteria, including the model bacterium *Bacillus subtilis* and various human pathogens. In *B*. *subtilis*, activation of Spx occurs in response to disulfide stress. We recently reported, however, that induction of Spx also occurs in response to cell wall stress, and that the molecular events that result in its activation under both stress conditions are mechanistically different. Here, we demonstrate that, in addition to up-regulation of *spx* transcription through the alternative sigma factor σ^M^, full and timely activation of Spx-regulated genes by cell wall stress requires Spx stabilization by the anti-adaptor protein YirB. YirB is itself transcriptionally induced under cell wall stress, but not disulfide stress, and this induction requires the CssRS two-component system, which responds to both secretion stress and cell wall antibiotics. The *yirB* gene is repressed by YuxN, a divergently transcribed TetR family repressor, and CssR~P acts as an anti-repressor. Collectively, our results identify a physiological role for the YirB anti-adaptor protein and show that induction of the Spx regulon under disulfide and cell wall stress occurs through largely independent pathways.

## Author summary

*Bacillus subtilis* Spx is the founding member of a large family of redox-stress sensing transcriptional regulatory proteins, and Spx orthologs are important for oxidative stress and virulence in several Gram-positive pathogens. Spx controls a large regulon in response to disulfide stress. Disulfide stress induces the Spx regulon through post-translational events that involve both stabilization of Spx against proteolysis and protein oxidation. We previously reported that genes in the Spx regulon are also induced in response to antibiotics that target the synthesis of the bacterial cell wall. Interestingly, we show that this induction is mechanistically distinct from disulfide stress as it involves transcriptional induction of *spx* by an alternative sigma factor. We show here that stabilization of Spx also requires a novel anti-adaptor protein, YirB, which prevents Spx degradation by binding to and inhibiting the activity of the adaptor protein YjbH. Induction of *spx* and Spx stabilization are both required for full and timely induction of the genes in the Spx regulon in response to cell wall stress. We further show that induction of the genes in the Spx regulon in response to either cell wall stress or disulfide stress takes place through largely independent pathways.

## Introduction

In its natural habitat, the soil-dwelling bacterium *Bacillus subtilis* is continuously exposed to stressful conditions that can compromise its survival. To adapt, bacteria must be able to sense the stress and respond accordingly. Adaptation to stress often requires the interplay of multiple signaling pathways and regulators. At the transcriptional level, gene expression is controlled by modulation of the activity of transcription factors, which through precise molecular interactions redirect the activity of RNA polymerase at specific sets of genes [1]. In *B*. *subtilis*, for example, the cell envelope stress response is mediated by the individual or coordinated action of extracytoplasmic (ECF) sigma factors (e.g. σ^Μ^, σ^W^, and σ^X^) [2], two-component signal transduction systems (e.g. LiaRS and BceSR) [3], and other transcription regulators (e.g. Spx) [2–4]. The activity of transcription factors can be regulated by changes in their expression or allosteric regulation of their activity. Adaptation to stress may also involve regulated proteolysis of transcription factors [5].

Proteolysis mediated by the Clp ATP-dependent proteases plays a critical role in regulation, as it permits the selective degradation of specific sets of proteins [5]. The proteins degraded by the Clp proteases generally contain a protein tag, which is recognized by either the protease itself or an adaptor and targets them for degradation [5]. When degradation requires an adaptor, the synthesis of an anti-adaptor protein can antagonize its activity, and allow the stabilization of the target protein [6–8]. In *B*. *subtilis*, for example, the proteolysis of the master regulator of competence ComK requires the adaptor protein MecA and the ClpCP protease. The presence of ComS, an anti-adaptor protein, allows ComK accumulation by interfering with the MecA-ComK interaction [8]. Also, in *Escherichia coli* and *Salmonella*, a set of anti-adaptors expressed under various environmental conditions (e.g. phosphate starvation, DNA damage, or magnesium starvation) permits stabilization, against ClpXP-mediated proteolysis, of the sigma factor RpoS through direct interaction with the adaptor RssB [6,7].

The Spx protein is a global regulator in *Bacillus subtilis*, and other low-GC Gram-positive bacteria (Phylum *Firmicutes*) [9–12]. Induction of the Spx regulon is best understood in the case of disulfide stress, but it is also noted under conditions that result in protein denaturation and misfolding (i.e. heat shock or ethanol stress) [9,13]. Recently, cell wall stress was also reported to trigger the induction of the Spx regulon [4]. Spx controls the expression of a large number of genes that help the cells to cope with stressful conditions, and includes genes involved in the synthesis of cysteine and bacillithiol, as well as the thioredoxin system, and the ATP-dependent Clp proteases [9,14,15]. While the functional role of the Spx regulon during disulfide and heat stress is fairly well understood [9,13], its role during cell wall stress is less clear.

A complex regulatory network drives the expression, stability, and activity of Spx. At the transcriptional level, the expression of *spx* is driven from at least three promoters controlled by different sigma factors: σ^B^, σ^M^, and σ^A^ [4,16–18]. The induction of the σ^M^-dependent promoter (i.e. P_M1_) is important for activation of the Spx regulon in response to cell wall stress [4], whereas expression of *spx* from the intergenic promoters is sufficient to complement an *Δspx* knockout mutant for diamide resistance [9,18]. The functional role of the σ^B^ promoter in the induction of the Spx regulon has not yet been defined. Additionally, the protein repressors PerR and YodB modulate the expression of *spx* in response to hydrogen peroxide and electrophilic compounds, respectively [19]. The activity of Spx is modulated by a redox-sensing switch (i.e. contains a CxxC motif) located at its N-terminus [20], which increases the activity of the protein when oxidized. Oxidation of Spx is, however, not required for the induction of all Spx-regulated genes [4,14,15], and thus the requirement for Spx oxidation seems to depend on the specific nature of the stress. It is still unknown, however, the extent to which the oxidation status of Spx impacts the composition of the regulon.

Although *spx* is highly transcribed in exponentially growing cells, Spx levels remain low due to active proteolysis [21]. Spx degradation occurs upon binding of the adaptor protein YjbH to a region near the Spx C-terminus, which targets the protein for degradation via the ATP-dependent protease ClpXP [22–24]. Under disulfide stress, the oxidation of YjbH and ClpX, as well as the aggregation of YjbH, result in a dramatic reduction in Spx proteolysis [22,23,25]: accumulation of Spx, along with the oxidation of its redox switch, then lead to activation of the regulon. Accumulation of Spx under cell wall stress, by contrast, largely depends on transcriptional up-regulation of *spx*, although post-transcriptional effects also appear to play a role [4].

Here we demonstrate that, in addition to transcriptional induction of *spx* by an alternative sigma factor (i.e. σ^M^) [4], stabilization of Spx is also required for full induction of the Spx regulon in response to cell wall stress. Interestingly, this stabilization is mediated by the YirB anti-adaptor protein, which is rapidly induced under conditions of cell wall stress but not disulfide stress. The expression of *yirB* itself is regulated by the coordinated action of both a two-component system (i.e. CssRS) and a TetR-like repressor (i.e. YuxN). Notably, we found that CssR~P activates the *yirB* promoter by acting as an anti-repressor of YuxN-mediated repression. Finally, we show that activation of the Spx regulon by cell wall stress and disulfide stress takes place through largely independent pathways, providing an example of orthogonality in signal transduction pathways. This study further expands the diversity of regulatory mechanisms known to govern induction of the Spx regulon in response to stress.

## Results

### A post-transcriptional event contributes to activation of Spx in response to cell wall stress

Previously we demonstrated that, unlike disulfide stress, induction of the Spx regulon in response to cell wall stress is driven by upregulation of the *spx* gene through a σ^Μ^-dependent promoter (i.e. P_M1_) [4]. Consistent with this, cells harboring a non-functional P_M1_ (i.e. P_M1_*) promoter display a dramatic decrease in both Spx accumulation and induction of Spx-controlled genes in response to cell wall active antibiotics [4]. Over the course of those experiments, however, we noted that even under conditions wherein *spx* cannot be induced, cell wall stress still led to a slight increase in the concentration of Spx and upregulation of Spx-controlled genes [4]. This suggested that Spx stabilization may also contribute to induction of the Spx regulon.

**Fig 1:**
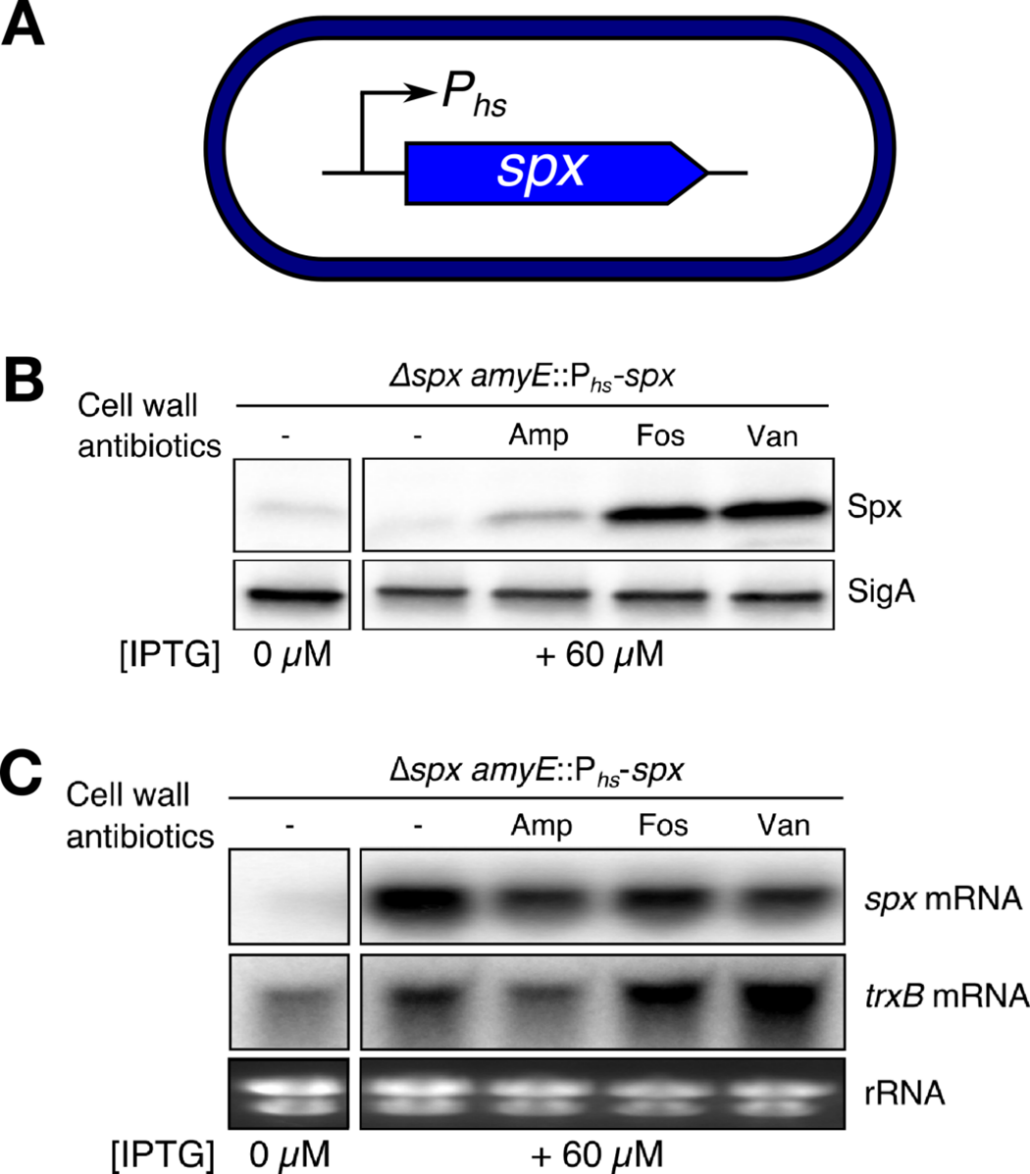
A post-transcriptional event contributes to activation of Spx in response to cell wall stress. (A) The *spx* gene was placed under control of IPTG as the only source of *spx* for the cells. (B) WT cells were grown with a fixed concentration of inducer and treated or not with 2 μg ml^-1^ ampicillin, 200 μg ml^-1^ fosfomycin, or 1 μg ml^-1^ vancomycin. Spx protein levels were monitored before and after 30 min of treatment using western blot. (C) The *spx* mRNA and *trxB* mRNA levels were simultaneously studied using northern blot. The blots are representative of at least two biological replicates. The “-” symbol indicates untreated cells.

To further define if stabilization is important under cell wall stress, we studied Spx accumulation in cells with conditional expression of *spx* from an IPTG-inducible promoter (i.e. P_*hs*_*-spx*) (Fig 1A). Since these cells are unable to induce *spx* transcription in response to cell wall antibiotics, an increase in Spx levels might reflect protein stabilization. As seen in the wild-type strain [4], treatment with different cell wall antibiotics elicited Spx accumulation in the conditional strain (Fig 1B), while the *spx* mRNA levels were not elevated upon antibiotic treatment (Fig 1C). Induction of the Spx-controlled gene *trxB* was also observed in response to fosfomycin and vancomycin, but not ampicillin (Fig 1C). In WT, induction of *trxB* in response to ampicillin is maximal after 20 min of treatment [4], which likely explains why no induction was observed here. Nevertheless, these results further support our hypothesis that cell wall stress results in increased Spx activity independent of transcriptional induction. Altogether, these observations suggest that both transcriptional and post-transcriptional mechanisms are important for induction of Spx-controlled genes in response to cell wall stress.

### The anti-adaptor protein YirB is required for Spx stabilization

**Fig 2:**
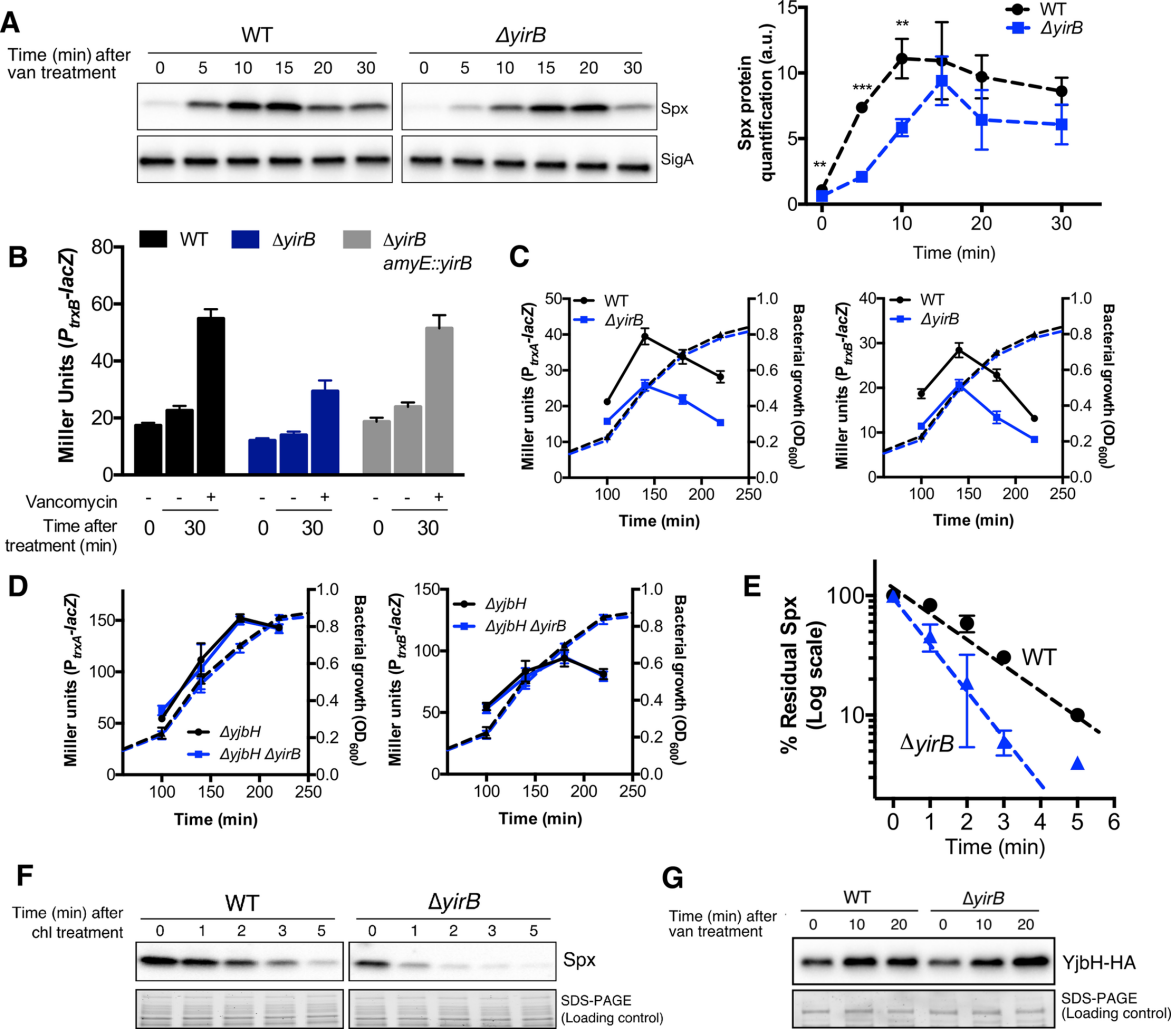
The anti-adaptor YirB is required for Spx stabilization. (A) Accumulation of Spx in response to vancomycin treatment was determined in a time-course experiment in WT and *ΔyirB* cells using western blot. A representative blot is shown in the left panel. Relative quantification of Spx protein levels in both strains is plotted on the right panel. Data were normalized using the Spx levels of the WT strain before induction as reference. Error bars represent SEM of three biological replicates. One, two, and three asterisks indicate significant differences with P < 0.05, P < 0.01 and P < 0.001 respectively, as estimated using the T-test. The statistical analysis to compare Spx levels in both strains was independently performed for every time point. (B) Expression levels (i.e. β-galactosidase activity) of the Spx-controlled gene *trxB* in WT, *ΔyirB*, and complementation strain (*ΔyirB + amyE*::*yirB)* following treatment or not with 1 μg ml^-1^ vancomycin. (C) Analysis of the activity of the *trxA* and *trxB* promoters in WT and *ΔyirB* during exponential and early stationary phase in LB medium (solid lines). The bacterial growth curves are also displayed on the figure (dashed lines) (D) Analysis of the activity of the *trxA* and *trxB* promoters in the strains *ΔyjbH* and *ΔyjbH ΔyirB*. (E)- (F) Effect of YirB on Spx stability during cell wall stress. The half-life of Spx was determined in exponentially growing cells treated or not with vancomycin. The percentage of remaining Spx was normalized with respect to time 0 min. (G) The concentration of YjbH-HA was determined by western blot in WT and Δ*yirB* cells. All experiments were performed in triplicate. Error bars indicate SEM.

When overexpressed, the YirB protein functions as an anti-adaptor protein that can inhibit the YjbH adaptor resulting in stabilization of Spx [26]. However, the physiological role of YirB has not been defined, and it is not important under diamide stress conditions [26]. We thus hypothesized that YirB is responsible for stabilization of Spx in response to cell wall stress. To determine if YirB affects Spx accumulation, we measured Spx protein levels in both WT and *ΔyirB* cells in response to vancomycin stress. Consistent with our hypothesis, deletion of YirB caused a decrease in the overall Spx levels, as well as a change in the dynamics of Spx accumulation: while in the WT strain the Spx protein was rapidly accumulated, cells lacking YirB displayed a significant delay in the accumulation of Spx (Fig 2A).

In the absence of YirB, Spx accumulated to generally lower levels (Fig 2A). This decrease in Spx levels was reflected in reduced expression of the Spx-dependent target gene *trxB* in response to vancomycin treatment (Fig 2B). As expected, ectopic complementation of the *yirB* null mutation restored the wild-type phenotype (Fig 2B). In the course of these studies, we also observed that cells lacking YirB displayed overall reduced *trxA* and *trxB* promoter activity during growth in LB medium (Fig 2C), suggesting that YirB also affects basal expression of Spx-controlled genes in growing cells. As expected, the impact of the deletion of *yirB* on both *trxA* and *trxB* was eliminated in cells lacking the adaptor protein YjbH (Fig 2D).

Since YirB is a putative anti-adaptor protein [26], we reasoned that cells lacking YirB should display reduced Spx stability. To test this idea, we treated log phase cells with vancomycin, incubated the cells for 10 min to allow accumulation of Spx (and potentially YirB), and monitored protein half-life in chloramphenicol treated cells by western blot (Fig 2E and 2F). Under these conditions, the half-life of Spx in WT was ~2 min, whereas in Δ*yirB* the half-life was reduced to < 1 min (Fig 2E and 2F). Importantly, the decrease in the stability of Spx in Δ*yirB* cells was not due to abnormally elevated YjbH levels. Indeed, we observed that deletion of YirB led to slightly lower levels of YjbH after 10 min of induction (i.e. when the Spx chase was carried out) (Fig 2G), which is consistent with the fact that *yjbH* is itself an Spx-controlled gene [4,14].

### YirB also stabilizes Spx in cells with conditional expression of *spx*

**Fig 3:**
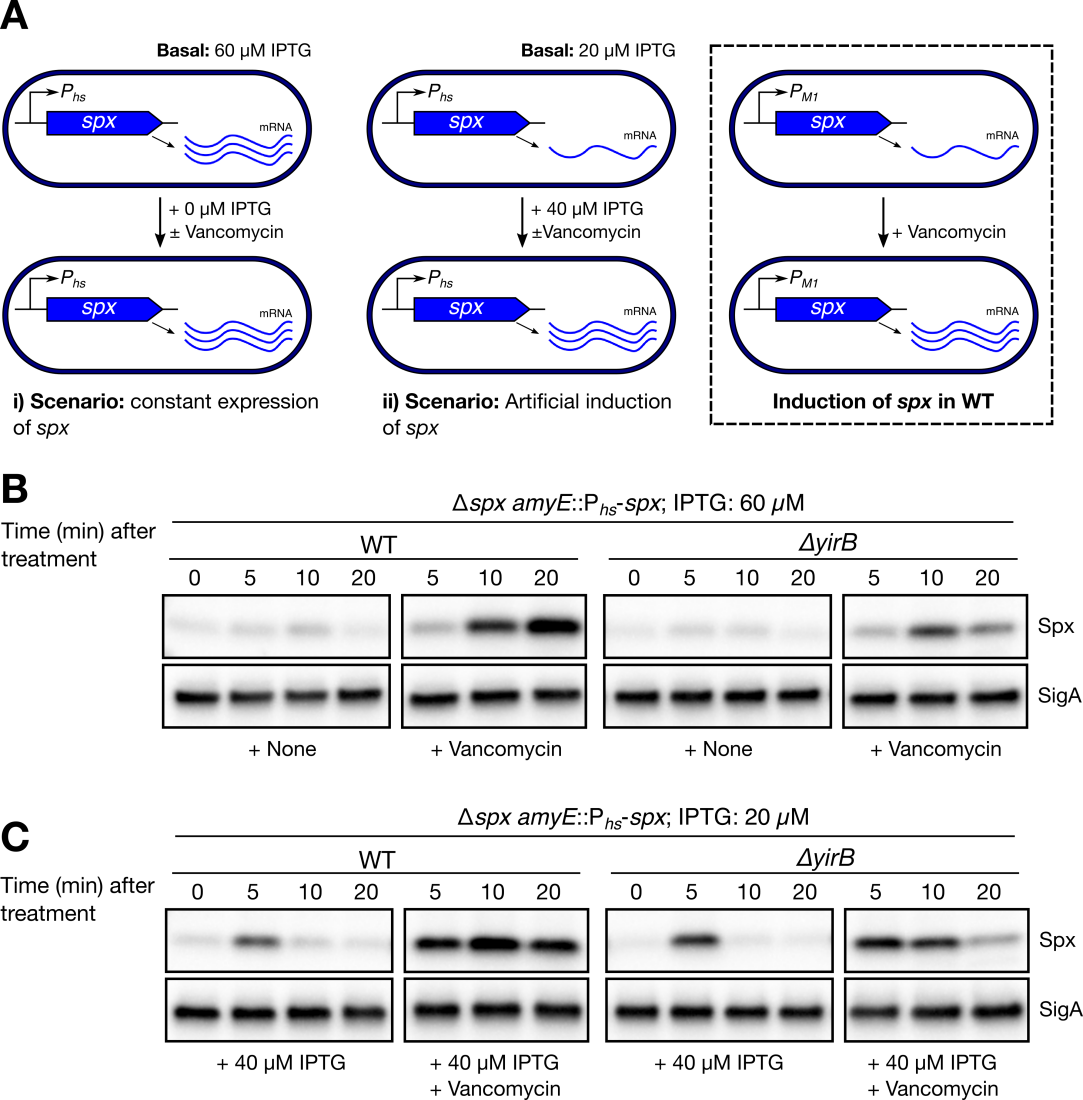
YirB also stabilizes Spx in cells with conditional expression of *spx*. (A) Graphical description of the experimental scenarios in Fig 3B and 3C. The regulation of *Spx* in wild-type cells was included for reference (right box). (B) Spx levels were monitored in WT and Δ*yirB* cells featuring conditional expression of *spx* (i.e. *spx* expression level was fixed using 60 μM IPTG, scenario i) using western blot. Cells were treated or not with 1 μg ml^-1^ vancomycin. (C) Spx levels were monitored in WT and Δ*yirB* cells featuring conditional expression of *spx* (i.e. *spx* expression was fixed using 20 μM IPTG, scenario ii) using western blot. At the same time, expression of *spx* was upregulated by adding inducer to achieve 60 μM IPTG and cells were treated or not with 1 μg ml^-1^ vancomycin. The blots presented are representative of three biological replicates, which produced similar results.

The *spx* gene is under exceptionally complex control, since several promoters (i.e. P_A_, P_M1_, P_M2_, and P_B_) [4,16–18] and repressors (i.e. PerR and YodB) [19] modulate its expression. Induction of *spx* in response to cell wall stress, for instance, is driven from P_M1_ as shown in Fig 3A (right box). In order to separate any potential effects of YirB on *spx* transcription and determine the actual contribution of YirB to Spx accumulation, we studied Spx dynamics in engineered WT and Δ*yirB* cells featuring conditional expression of *spx* (Fig 3A). When cells were grown in the presence of a fixed concentration of inducer (i.e. LB medium + 60 μM IPTG; Fig 3A, scenario i), we observed that accumulation of Spx occurred in a vancomycin-dependent fashion (Fig 3B, left two panels), as previously seen (Fig 1B). Deletion of *yirB* reduced, but did not completely eliminate, the vancomycin-induced accumulation of Spx (Fig 3B). Deletion of *yirB* also affected induction of *trxB* (S1A Fig). These results suggest that YirB-dependent stabilization of Spx (Fig 2E and 2F) is important for accumulation of Spx and activation of its regulon in response to cell envelope stress.

Under cell wall stress the expression of *spx* is dynamic since the P_M1_ promoter is induced in response to antibiotic treatment (Fig 3A, right box). To assess the contribution of YirB under conditions wherein *spx* is induced, we studied the effect of artificial induction of *spx* on Spx levels in the engineered WT and Δ*yirB* cells (Fig 3A, scenario ii). For this, cells were grown in LB broth + 20 μM IPTG (i.e. basal induction levels) and then treated with inducer to reach 60 μM IPTG, thereby mimicking the effect of antibiotic induction of *spx* from the P_M1_ promoter. In the absence of vancomycin, addition of inducer resulted in only a transient accumulation of Spx (Fig 3C, first panel), and this effect was independent of YirB. This suggests that induction of *spx* from P_hs_ is sufficient for transient accumulation of Spx protein, and the concomitant induction of the σ^Μ^ regulon is not required. However, much stronger and long-lasting induction of Spx was observed if there was both an increase in *spx* transcription and antibiotic treatment (Fig 3C, left two panels). Deletion of *yirB* resulted in minor differences between WT and Δ*yirB* under unstressed conditions; however, cell wall stress resulted in increased Spx accumulation and *trxB* induction in a YirB-dependent fashion (Fig 3C and S1B). Altogether, these results show that YirB stabilizes Spx under cell wall stress, and that activation of *spx* transcription and Spx stabilization are additive. Further, they suggest that stabilization of Spx can still occur in a YirB-independent fashion.

### Both induction of *spx* and Spx stabilization are required for full and timely induction of Spx-controlled genes in response to cell wall stress

**Fig 4:**
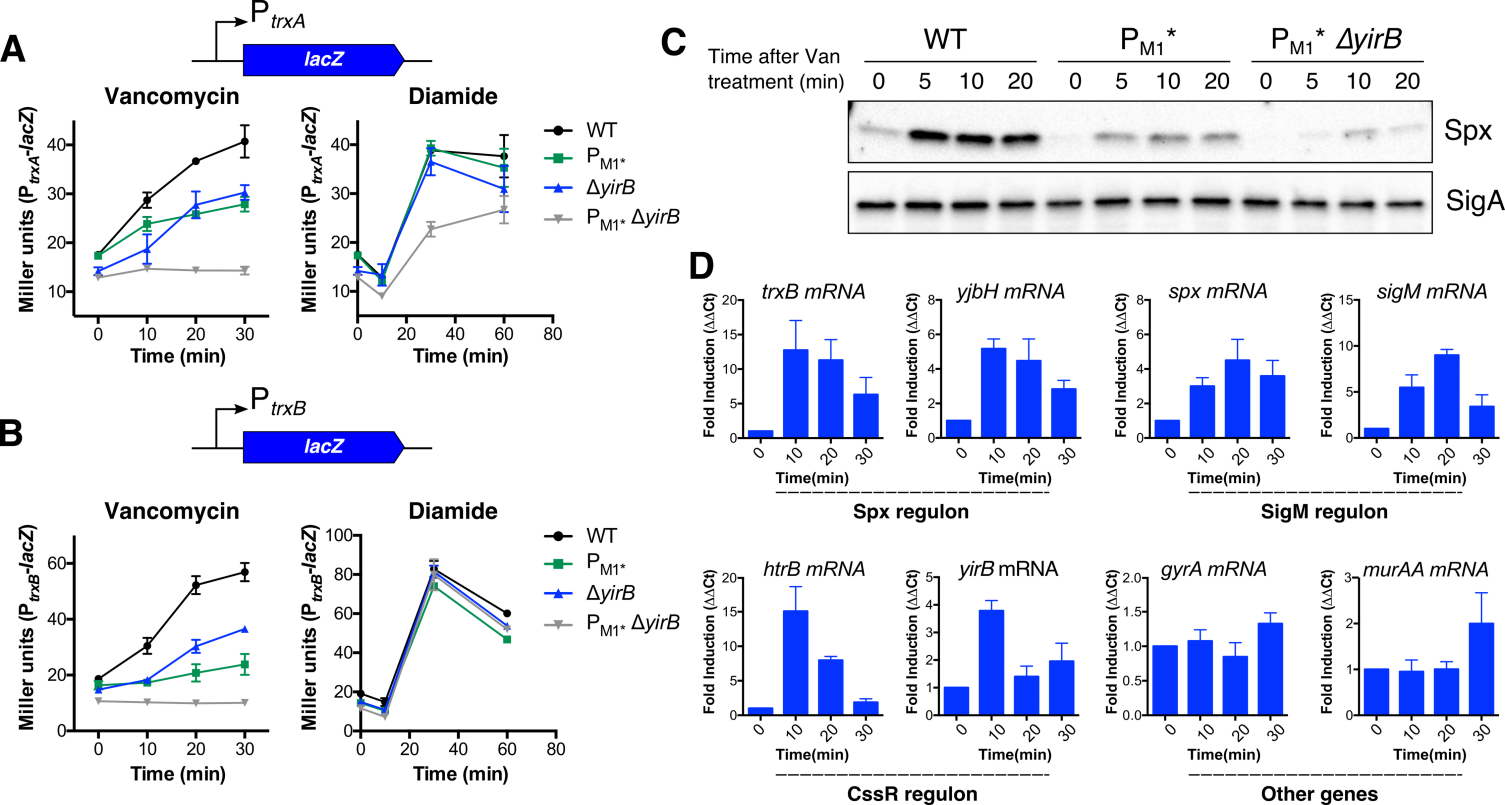
Both induction of *spx* and Spx stabilization are required for full and timely induction of Spx-controlled genes in response to cell wall stress. **Activation of Spx by cell wall and disulfide stress takes place through independent pathways.** The expression dynamics of the genes (A) *trxA* and (B) *trxB* were monitored in WT, *P*_M1_*, *ΔyirB*, and the double mutant *P*_M1_* ΔyirB. Cells were treated with vancomycin or diamide (N = 4). (C) Spx levels in WT, *P*_M1_*, and *P*_M1_* ΔyirB after treatment with vancomycin revealed additivity between transcriptional induction of *spx* and Spx stabilization (N = 4), as determined using western blot. D) Expression profile of selected genes using RT-qPCR (N = 3). The mRNA levels were normalized against the 23S rRNA. Error bars represent SEM.

While accumulation of Spx in response to disulfide stress relies on reduced proteolysis [22,23,25], Spx accumulation in response to cell wall stress is more complex as it requires both σ^Μ^-dependent *spx* upregulation [4] and YirB-mediated Spx stabilization. To study how both transcriptional induction and stabilization together contribute to activation of the Spx regulon, we monitored the induction of the *trxA* and *trxB* genes using *lacZ* transcriptional fusions in WT, Δ*yirB*, P_M1*_, and Δ*yirB* P_M1*_ cells. Deletion of *yirB* or inactivation of P_M1_ (i.e. P_M1*_) led to a significant decrease in the induction of both fusions. Furthermore, in the Δ*yirB* P_M1*_ double mutant the *trxA* and *trxB* genes were no longer responsive to vancomycin treatment (Fig 4A and 4B). Assessment of the protein levels also provided evidence of additivity, which was more noticeable early after induction (Fig 2A and Fig 4C). These results demonstrate that both transcriptional induction and stabilization are required for full induction of Spx-controlled genes in response to cell wall stress. They also show that the previously observed induction of Spx-regulated genes in absence of P_M1_ [4] was largely due to YirB.

Our time course studies (Fig 2A) suggested that YirB is important for the rapid accumulation of Spx early after antibiotic treatment. In support of this, we also noted that cells lacking YirB, yet still able to induce P_M1_, displayed a delay in induction of *trxA* and *trxB* in response to vancomycin treatment (Fig 4A and 4B). Furthermore, using RT-qPCR, we noticed that whereas the expression of *trxB* and *yjbH* (i.e. two Spx-controlled genes) was strongly and rapidly induced, with maximal expression 10 min after treatment, σ^M^-dependent induction of *spx* and the autoregulated *sigM* gene was not maximal until 20 min after treatment (Fig 4D). The observed dynamics of both accumulation of Spx and induction of Spx-controlled genes therefore reflect both protein stabilization by YirB (most important early after vancomycin treatment) and increased transcription of *spx* (most important at later times).

### Induction of the Spx regulon in response to cell wall stress and disulfide stress takes place through independent pathways

Our current and previous findings [4] suggest that induction of the Spx regulon in response to cell wall stress and disulfide stress may occur through fully independent pathways. First, transcriptional induction of *spx* is only important under cell wall stress conditions [4]; second, unlike disulfide stress, the redox-sensing switch plays a limited role in induction of Spx-controlled genes in response to cell wall-active antibiotics [4]; and third, YjbH aggregation, which is critical for Spx accumulation under disulfide stress, seems to play no role in Spx stabilization under vancomycin treatment (S2 Fig). Instead, the anti-adaptor protein YirB stabilizes Spx against proteolysis (Figs 2 and 3). To further determine whether the activation of the Spx regulon occurs through independent pathways, we studied the induction dynamics of *trxA* and *trxB* in response to disulfide stress in cells lacking *yirB* (and/or P_M1_) (Fig 4A and 4B). Remarkably, in cells treated with diamide, deletion of YirB (or P_M1_, as expected [4]) had no effect on induction of both *trxA* and *trxB*. Likewise, inactivation of both YirB and P_M1_ had no effect on the responsiveness of both fusions to disulfide stress (Fig 4A and 4B). We noted, however, a slight decrease in the induction of *trxA* in the *ΔyirB* P_M1_* strain (Fig 4A). Altogether, the present evidence suggests that activation of Spx in response to disulfide and cell wall stress takes place through orthogonal pathways (Fig 4A and 4B).

### Vancomycin induces *yirB* through the CssRS two-component system

**Fig 5:**
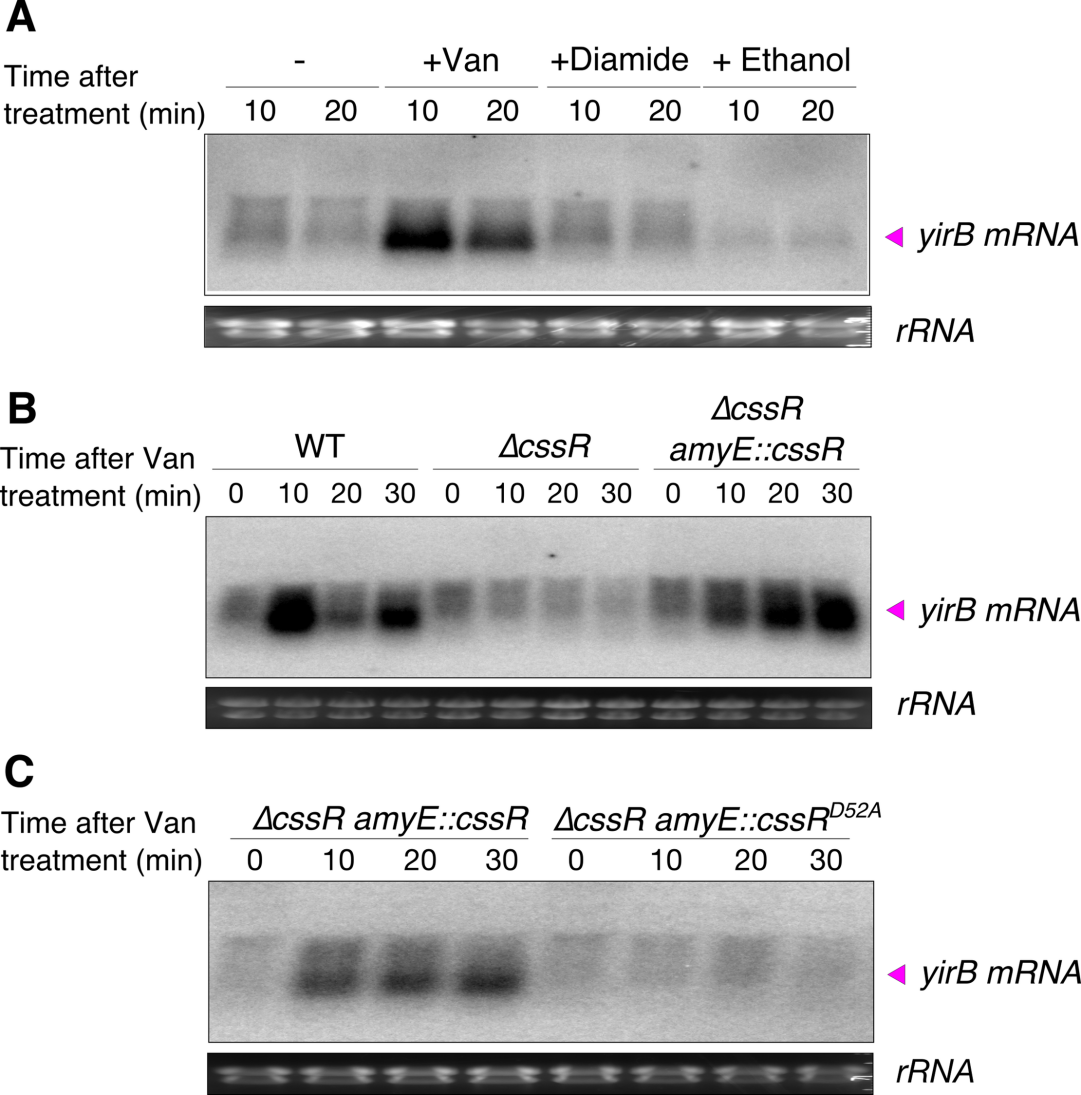
Vancomycin induces *yirB* through the CssRS two-component system. (A) The *yirB* transcript (~300 nt) is induced under cell wall stress, but not disulfide or ethanol stress. Total RNA was isolated from WT cells treated or not with 1 μg ml^-1^ vancomycin, 500 μM diamide, and 5% ethanol, and *yirB* mRNA levels studied by northern blot. The “-” symbol indicates untreated cells. (B) Induction of *yirB* requires the two-component system CssRS. Transcriptional profile of *yirB* in WT, *ΔcssR*, and the complementation strain (*ΔcssR + amyE*::*cssR*) after treatment with 1 μg ml^-1^ vancomycin as determined by northern blot. (C) Induction of *yirB* by CssR, under vancomycin-induced stress, requires the phosphorylation of the Asp52 residue. The expression of *yirB* was studied by northern blot in Δ*cssR* cells complemented ectopically with either *cssR* or *cssR*^D52A^ from the *cssR* native promoter. The presented blots are representative of three independent experiments.

We next sought to determine how YirB activity might itself be regulated. First, we monitored *yirB* mRNA levels under conditions known to induce the Spx regulon including vancomycin (cell wall stress), diamide (disulfide stress), and ethanol treatment [9,25]. Remarkably, only vancomycin treatment resulted in a significant induction of *yirB*, which suggests that the role of YirB is specific to the cell wall stress response (Fig 5A). This may also explain why no differences in induction of the Spx regulon were previously found between WT and *ΔyirB* under disulfide stress [26].

The *yirB* gene is located downstream of *cssRS* and divergently transcribed from *yuxN* (see below). The *cssRS* genes encode a two-component system (TCS) that is known to respond to secretion stress [27], and *yuxN* encodes a putative repressor in the TetR family with yet unknown activity. We reasoned that since *yirB* and *cssRS* are genetic neighbors, the CssRS TCS might regulate *yirB* under cell wall stress. This hypothesis is supported by the fact that up-regulation of two CssRS-controlled genes (*htrA* and *htrB*) has been previously noted in response to cell wall stress [28,29]. Moreover, a global transcriptomic study of *B*. *subtilis* cells growing under a variety of conditions showed that the expression of *yirB* is highly correlated with *htrB*, which is a divergently transcribed gene regulated by the *cssRS* TCS [30]. Consistent with our hypothesis, cells lacking CssR were unable to induce *yirB* upon treatment with vancomycin; ectopic complementation of CssR fully restored the WT phenotype (Fig 5B). Furthermore, point mutations that replaced the conserved aspartic acid in the phosphorylation site of CssR by the amino acid alanine (i.e. CssR^D52A^) completely prevented induction of *yirB* following vancomycin treatment, further suggesting that the CssRS two-component system is responsible for upregulation of *yirB* in response to cell wall stress (Fig 5C). Since YirB seems to be most important for the increased accumulation of Spx early after antibiotic stress (Fig 4), we hypothesized that the CssRS system would be induced rapidly after antibiotic challenge. Indeed, both *yirB* and *htrB* mRNAs accumulated rapidly after vancomycin challenge (Fig 4D and Fig 5).

### CssR~P induces *yirB* expression by antagonizing YuxN repression

**Fig 6:**
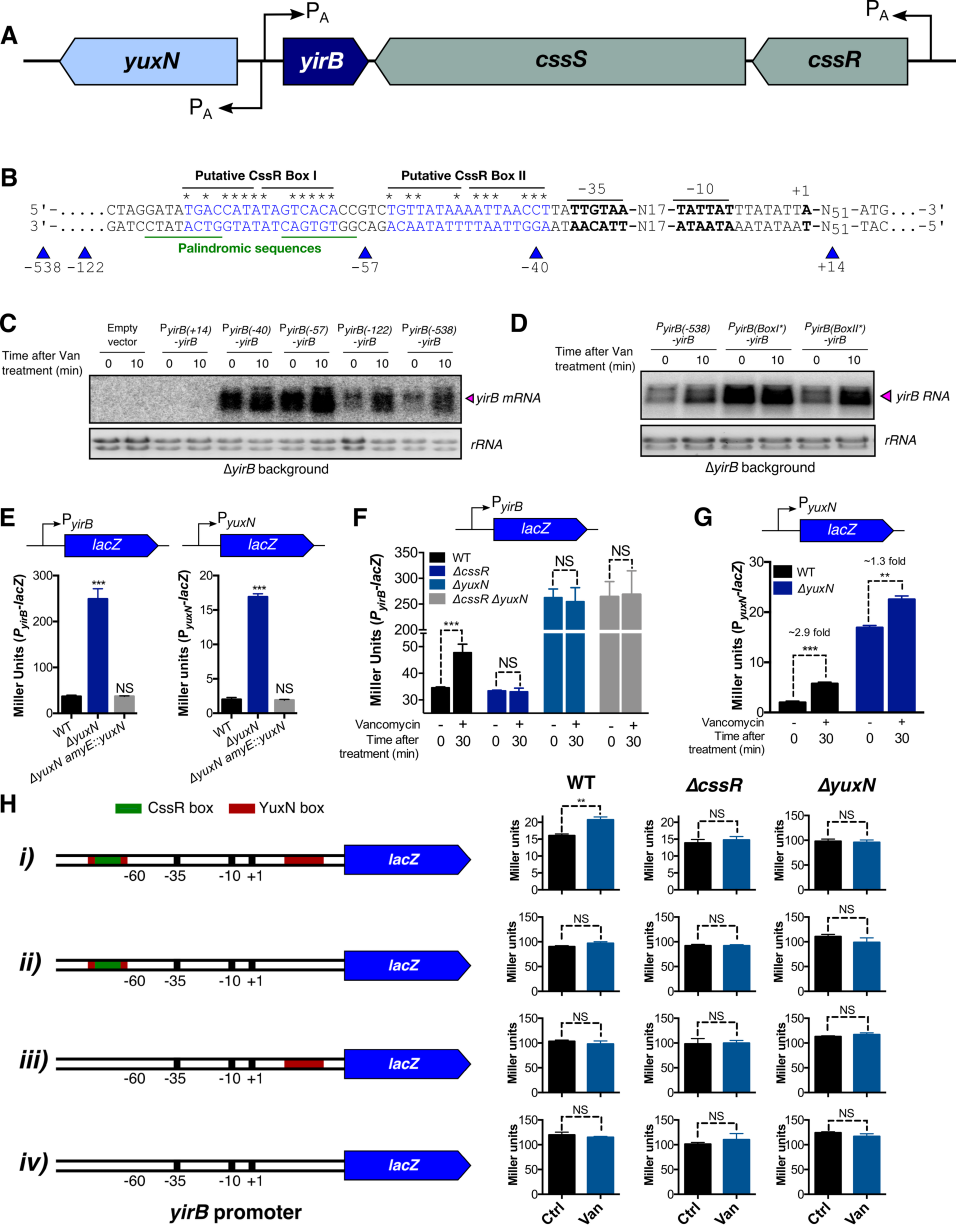
CssR~P induces *yirB* expression by antagonizing YuxN repression. (A) Genetic context of the *yirB* gene. (B) The transcription start site was mapped using 5’ RACE, and the identification of the putative -10 and -35 boxes was performed manually. Two putative CssR boxes were located upstream the -35 box, which exhibited similarity with the consensus CssR binding sequences (S3 Fig). (C) Promoter truncation analysis was used to determine the contribution of the upstream DNA sequences on *yirB* regulation. For this, the *yirB* gene along with the promoter truncations were integrated at the *amyE* site and used to complement a Δ*yirB* mutant. The positions were mapped with respect to the +1 site as shown in Fig 6B. The *yirB* mRNA levels were determined by northern blot. (D) The mRNA *yirB* levels were studied by northern blot in cells harboring the mutant CssR BoxI* (5’-TGACttTtTAGatAtt-3’) or CssR BoxII* (5’-aGaaATAAAATTAAaC-3’), and compared with P_*yirB(-538)*_*-yirB*. The three *yirB* promoter regions are identical except for the point mutations. The lower-case letters indicate the sites were the mutations were introduced (see Fig 6B). (E) Analysis of the P_*yirB*_-*lacZ* and P_*yuxN*_-*lacZ* reporter fusions in cells lacking YuxN. (F) Activity of the *yirB* promoter in WT, as well as in the *ΔcssR*, *ΔyuxN*, and *ΔcssR ΔyuxN* knockout mutants. (G) The *yuxN* gene is upregulated in response to cell wall stress. (H) Analysis of the *yirB* promoter featuring truncations in the YuxN boxes. The different promoters were fused to the *lacZ* gene and its activity measured in WT, *ΔcssR*, and *ΔyuxN* cells before and after 20 min of treatment with 1 μg ml^-1^ vancomycin. Error bars represent SEM of at least three independent replicates. One, two, and three asterisks indicate significant differences with P < 0.05, P < 0.01 and P < 0.001 respectively, as estimated using one-way ANOVA and the Tukey’s HSD test for Fig 6E, and the T-test for Fig 6F–6H. NS indicates no significant differences.

To further characterize the role of CssR in regulation of *yirB* we isolated RNA from vancomycin treated cells and used 5’ RACE to define the transcription start site. Transcription initiates 51 nt upstream of the start codon, and two putative CssR boxes [31] were apparent just upstream of the -35 region (Fig 6A and 6B, S3 and S4 Figs). We first used promoter truncations (i.e. P_*yirB(x)*_-*yirB*) to monitor the effect of upstream sequences on *yirB* mRNA levels (Fig 6B and 6C). Interestingly, truncations that contained either only the RNA polymerase (RNAP) binding-site [i.e. P_*yirB(-40)*_] or both the RNAP binding-site and putative CssR BoxII [i.e. P_*yirB(-57)*_] displayed high *yirB* mRNA basal levels and were unresponsive to cell wall stress. The inclusion of predicted BoxI [i.e. P_*yirB(-122)*_ and P_*yirB (-538)*_] sufficed to restore the WT phenotype: a low basal level and induction by vancomycin (Fig 6B). Next, we introduced point mutations to disrupt the most conserved positions in BoxI or BoxII. Point mutations in BoxI rendered *yirB* mRNA basal levels high and unresponsive to vancomycin, while point mutations in the predicted CssRS BoxII had little effect (Fig 6D). The activity of *trxB* was also affected by the mutations in P_*yirB*_ (S5 Fig).

Since CssR is required for induction of *yirB* (Fig 5), we initially hypothesized that CssR would bind to CssR BoxI to activate transcription. However, our promoter truncation analysis reveals that P_*yirB*_ is highly active in cells in which BoxI is deleted (Fig 6B and 6C), and this is supported by the effect of point mutations (Fig 6D). These observations imply that BoxI is itself a negative regulatory element for P_*yirB*_ activity. Analysis of this DNA region in *B*. *subtilis*, as well as other *Bacillus* species (S4 Fig), revealed the presence of a conserved palindromic sequence that lies on top of the predicted CssR BoxI (Fig 6B and S4). Likewise, we noted that a similar palindromic region is present in between positions +6 and +28 relative to P_*yirB*_; this palindrome overlapped the divergent *yuxN* promoter (S4 Fig). These observations suggest a model in which YuxN, a TetR-like repressor, binds to this palindromic sequence as a repressor, both for *yirB* and its own transcription. The role of CssR in this system could then be as an anti-repressor to alleviate the YuxN-dependent repression by binding to the overlapping CssR BoxI.

To test this model, we explored the role of YuxN in regulating expression of both *yirB* and *yuxN*. For this, we first studied whether YuxN binds the palindromic sequences. We reasoned that if YuxN binds the palindromes, deletion of YuxN would have no effect on expression from constructs lacking these DNA boxes. Using *lacZ* transcriptional fusions of truncated P_*yirB*_ (as shown in Fig 6H), we found this to be the case: deletion of YuxN in WT led to a dramatic increase in beta-galactosidase activity when the promoter contained both DNA boxes (i.e. see promoter *i*), however *yuxN* deletion had virtually no effect on the *yirB* promoters lacking one or both palindromes. Thus, we conclude that the palindromes are indeed YuxN boxes and that these two sites function cooperatively (Fig 6H).

Next, we studied whether YuxN regulated the expression of *yirB* and/or *yuxN* itself. Deletion of YuxN resulted in a dramatic increase in the basal levels of both *yirB* and *yuxN* expression, and ectopic complementation restored the WT phenotype (Fig 6E). These results are consistent with YuxN acting as a repressor of both *yirB* and *yuxN*. If CssR functions as an anti-repressor for YuxN, then deletion of *cssR* should have no effect in a strain lacking YuxN. Indeed, this is the case since P_*yirB*_ is both fully derepressed and unresponsive to vancomycin in the Δ*cssR* Δ*yuxN* strain, as seen for the Δ*yuxN* mutant (Fig 6F). The cooperative role of the two YuxN boxes in mediating repression of *yirB* (Fig 6H) leads us to speculate that YuxN may form a repression loop that prevents access of RNA polymerase to both the *yirB* and *yuxN* promoters. Binding of CssR~P to CssR BoxI likely prevents YuxN binding to the overlapping binding site, alleviates YuxN repression and allows transcription. This model also explains the induction of *yuxN* in response to cell wall stress (Fig 6G), even though no apparent CssR binding sites are located upstream of P_*yuxN*_ (S4 Fig).

## Discussion

The accumulation of Spx and the induction of its regulon in response to disulfide stress occurs through reduced proteolysis [22,23,25,32]. Proteolysis is regulated by 1) oxidation and aggregation of the adaptor protein YjbH [22,25] and 2) a decrease in proteolytic activity of the ClpXP protease [32]. In response to cell wall stress, and unlike disulfide stress, transcriptional induction of *spx* takes place and is required for maximal accumulation of Spx and induction of the regulon [4]. Once the Spx protein is accumulated it primarily remains in the reduced state; reduced Spx is then capable of modulating transcription [4]. Notably, we observed that although transcriptional induction is critical for cell wall stress induction of the Spx regulon, a post-transcriptional event was also implicated in this response (Fig 1) [4]. Here we report that, in addition to transcriptional control [4], Spx stabilization against ClpXP-mediated proteolysis is also required for full and timely induction of Spx-controlled genes in response to cell wall stress. Remarkably, we found that, unlike disulfide stress, this stabilization during cell wall stress is mediated by the anti-adaptor protein YirB.

YirB was originally identified, through a yeast two-hybrid screen for YjbH-interacting proteins, as a small basic protein that was able to modulate Spx protein levels when artificially overexpressed [26]. YirB was found to modulate Spx levels through direct binding to the adaptor protein YjbH, which resulted in reduced binding of YjbH with Spx and therefore reduced ClpXP-mediated Spx proteolysis [26]. Although YirB bound YjbH with high affinity, and its overexpression significantly increased the stability of Spx, YirB did not affect Spx accumulation in response to diamide treatment. This suggested that YirB was likely important under other stress conditions. Cell wall stress indeed provides such a condition, as the regulatory mechanisms that result in induction of the Spx regulon in response to cell wall antibiotics display remarkable differences relative to disulfide stress [4]. Analysis of cells with conditional or native control of *spx* indeed showed that cells lacking YirB display reduced accumulation of Spx under both cell wall stress and active growth (Figs 2, 3 and 4).

The *yirB* gene lies upstream of the *cssRS* two-component system, and divergent from a gene encoding a putative transcription factor YuxN, a repressor protein of the TetR family. The genetic proximity between *yirB* and *cssRS*, as well as the correlation in the expression database between *htrB*, a CssRS-controlled gene, and *yirB* rendered the CssRS TCS as an attractive candidate for regulation of *yirB* under cell wall stress [30]. Genetic and transcriptomic analyses of the expression of *yirB* revealed that CssRS is indeed required for the transcriptional induction of *yirB* under cell wall stress (Fig 5). Additionally, we found that YuxN represses *yirB*, and CssR~P appears to be required as an anti-repressor to antagonize YuxN (Fig 6). In agreement with previous findings [26], diamide treatment did not lead to induction of the *yirB* gene, nor did deletion of *yirB* have a significant impact on the induction of *trxB* in the presence of diamide (Fig 4), suggesting that the stabilization of Spx mediated by YirB represents a hallmark of cell wall stress.

The CssRS TCS has shown to be induced by hypersecretion of soluble proteins such as the α-amylase, and therefore has been long associated to protein secretion stress [27,33]. The specific molecular signals that lead to its induction, however, are not yet fully understood [33]. Interestingly, cell wall stress also led to induction of CssRS, as upregulation of *htrB* and *yirB* took place following vancomycin treatment. Previous transcriptomic studies also revealed induction of *htrB* in response to cell wall stress [28,29]. We hypothesize that two events might potentially result in induction of CssRS under cell wall stress. First, the induction of regulons such as σ^M^, σ^W^, or LiaRS (which include several lipoproteins and membrane proteins) might lead to secretion stress. Indeed, mutants lacking σ^W^ displayed reduced induction of the CssRS regulon, however mutants lacking σ^M^ or LiaR exhibited increased CssRS activity (S6 Fig). Alternatively, protein aggregation might occur as a direct effect of cell wall damage under antibiotic treatment. Further studies are required to unveil the underlying mechanisms. Induction of Spx may be advantageous under secretion stress since Spx controls the expression of protein chaperones and proteases [14].

**Fig 7:**
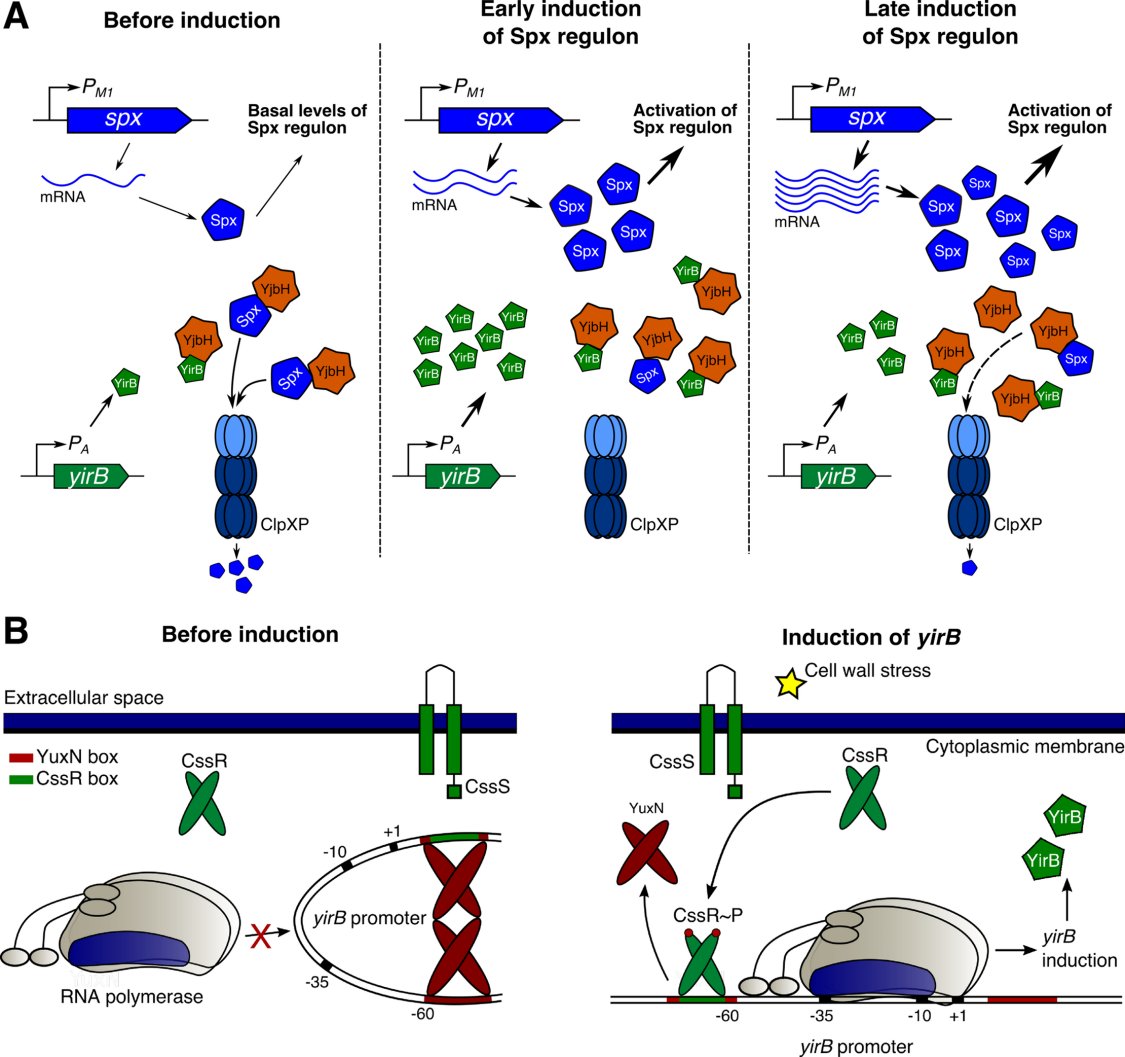
Model of regulation of Spx and *yirB* under cell wall stress. (A) Model of induction of the Spx regulon under cell wall stress. In exponentially growing cells (before induction), *spx* is constitutively expressed but Spx is actively degraded by the coordinated action of YjbH (i.e. the adaptor protein) and ClpXP (i.e. the protease). A fraction of Spx, however, remains stable at least in part due to the activity of YirB. This fraction of Spx, which is partially oxidized [4], drives the expression of Spx-controlled genes. After treatment with cell wall antibiotics (i.e. early induction), σ^M^ activates the expression of *spx* through the P_M1_ promoter, and CssR~P activates transcription of *yirB*. YirB then plays an important role in the stabilization of the newly synthesized Spx. Later in the process (i.e. late induction), induction of *spx* through P_M1_ is maximal, which leads to an increase in the amount of newly synthesized Spx. At the same time, the expression of *yirB* and presumably its role in stabilization decreases. The accumulation of Spx leads to induction of the genes in its regulon. (B) Model for regulation of *yirB*. The protein YuxN (here presented as a dimer for simplicity) binds the YuxN boxes upstream and downstream the *yirB* promoter forming a DNA loop. Upon treatment with cell wall antibiotics, the CssR protein becomes phosphorylated by the CssS histidine kinase. CssR~P then binds the CssR BoxI, which overlaps the YuxN box, leading to derepression of the *yirB* promoter. The synthesized YirB protein binds YjbH and prevents Spx proteolysis.

The induction of the Spx regulon in response to cell wall stress in *B*. *subtilis* thus involves the timely expression of *spx* itself by σ^Μ^ (an ECF sigma factor) and the anti-adaptor *yirB* by CssRS (a two-component system) (Fig 7). YirB is more important for early induction of the regulon, while upregulation of *spx* appears to be more important in later stages (Fig 7). Although the role of Spx in adaptation to cell wall antibiotics remains undefined, this study provides further evidence of the regulation mechanisms that control its induction. Importantly, the regulatory mechanisms that govern the induction of the Spx regulon in response to cell wall stress and disulfide stress take place through largely independent pathways, and thus provide a notable example of orthogonality in signal transduction systems. Our findings suggest a critical role of YirB in the activation of the Spx regulon; however, accumulation of Spx still occurs in cells lacking YirB (Figs 3 and 4), suggesting that further mechanisms are at play.

## Materials & methods

### Bacterial strains and culture conditions

All bacterial strains are listed in Table 1. *Bacillus subtilis* strains (all based on the *B*. *subtilis* 168 wild-type) were grown under standard conditions: lysogeny broth (LB) (10 g tryptone, 5 g yeast extract and 5 g NaCl per liter) broth at 37°C with vigorous shaking, unless otherwise stated. *Escherichia coli* DH5α was used for plasmid construction. Antibiotics were added to the growth medium when appropriate: 100 μg ml^-1^ ampicillin for *E*. *coli*, and 1 μg ml^-1^ erythromycin plus 25 μg ml^-1^ of lincomycin (MLS, macrolide-lincomycin-streptogramin B resistance), 10 μg ml^-1^ chloramphenicol, 100 μg ml^-1^ spectinomycin, and 10 μg ml^-1^ kanamycin for *B*. *subtilis*.

**Table 1:** Strains used in this study.

Number	Genotype* (phenotype)
HB18501	168 *trpC2* (WT)
HB18801	*spx*::*kan*
HB18504	*ΔyirB*
HB18506	*ΔcssR*
HB23044	*ΔyuxN*
HB23078	*ΔyuxN ΔcssR*
HB18905	*spx*::*P*_spx(PM1*)_*-yjbC-spx (kan)*
HB18903	*spx*::*P*_*spx(wt)*_*-spx*
HB18658	*thrC*::P_*trxB*_-*lacZ* (*ery)*
HB23089	*thrC*::*P*_*trxA*_*-lacZ (ery)*
HB18524	*thrC*::*P*_*yirB*_*-lacZ (ery)*
HB23091	*thrC*::*P*_*yuxN*_*-lacZ (ery)*
HB18805	*spx*::*kan amyE*::*P*_*spac*_*-spx (spec)*
HB18595	*spx*::*kan amyE*::P_*spac*_-*spx* (*spec*) *thrC*::P_*trxB*_-*lacZ* (*ery*)
HB18571	*ΔyirB spx*::*neo amyE*::P_spac_-*spx* (*spec*) *thrC*::P_*trxB*_-*lacZ* (*ery*)
HB18510	*ΔyirB thrC*::P_*trxB*_-*lacZ* (*ery*)
HB18521	*ΔyirB amyE*::*P*_*yirB(-538*, *+51)*_*- yirB (cm) thrC*::P_*trxB*_-*lacZ* (*ery*)
HB18554	*ΔcssR amyE*::*cssR (cm)*
HB18564	*ΔcssR amyE*::*cssR*^*D52A*^ *(cm)*
HB23030	*ΔyirB amyE*::*P*_*yirB(+14*, *+51)*_*-yirB (cm) thrC*::P_*trxB*_-*lacZ* (*ery)*
HB23031	*ΔyirB amyE*:: *P*_*yirB(-40*, *+51)*_*-yirB (cm) thrC*::P_*trxB*_-*lacZ* (*ery)*
HB23032	*ΔyirB amyE*::*P*_*yirB(-57*, *+51)*_*-yirB (cm) thrC*::P_*trxB*_-*lacZ* (*ery)*
HB23033	*ΔyirB amyE*::*P*_*yirB(-122*, *+51)*_*-yirB (cm) thrC*::P_*trxB*_-*lacZ* (*ery*)
HB23016	*ΔyirB amyE*::*cm thrC*::P_*trxB*_-*lacZ* (*ery*)
HB23015	*ΔyirB amyE*::*P*_*yirB(BoxI)*_*-yirB (cm) thrC*::*P*_*trxB*_*-lacZ (ery)*
HB18577	*ΔyirB amyE*::*P*_*yirB(BoxII)*_*-yirB (cm) thrC*::*P*_*trxB*_*-lacZ (ery)*
HB18567	*ΔyirB thrC*::*P*_*trxB*_*-lacZ (ery) spx*::*P*_*spx(wt)*_*-spx (kan)*
HB18568	*ΔyirB thrC*::*P*_*trxB*_*-lacZ (ery) spx*::*P*_spx(ΔPM1*)_*-spx (kan)*
HB18569	*thrC*::*P*_*trxB*_*-lacZ (ery) spx*::*P*_*spx(wt)*_*-spx (kan)*
HB18570	*thrC*::*P*_*trxB*_*-lacZ (ery) spx*::*P*_spx(ΔPM1*)-_*spx (kan)*
HB18588	*yjbH-HA (spec)*
HB23008	*ΔyirB yjbH-HA (spec)*
HB23132	*ΔyirB thrC*::*P*_*trxA*_*-lacZ (ery)*
HB23080	*ΔcssR thrC*::*P*_*yirB*_*-lacZ (ery)*
HB23082	*ΔyuxN thrC*::*P*_*yirB*_*-lacZ (ery)*
HB23084	*ΔyuxN ΔcssR thrC*::*P*_*yirB*_*-lacZ (ery)*
HB23079	*ΔcssR thrC*::*P*_*trxB*_*-lacZ (ery)*
HB23081	*ΔyuxN thrC*::*P*_*trxB*_*-lacZ (ery)*
HB23083	*ΔyuxN ΔcssR thrC*::*P*_*trxB*_*-lacZ (ery)*
HB23077	*ΔyuxN thrC*::*P*_*yirB*_*-lacZ (ery) amyE*::*yuxN (cm)*
HB23092	*ΔyuxN thrC*::*P*_*yuxN*_*-lacZ (ery)*
HB23136	*ΔyuxN amy*::*yuxN (cm) thrC*::*P*_*yuxN*_*-lacZ (ery)*
HB23133	*spx*::*P*_spx(PM1*)_*-yjbC-spx (kan) thrC*::*P*_*trxA*_*-lacZ (ery)*
HB23134	*ΔyirB spx*:*P*_spx(PM1*)_*-yjbC-spx (kan) thrC*::*P*_*trxA*_*-lacZ (ery)*
HB23171	*WT thrC*::*P*_*yirB(-128*, *+46)*_*-lacZ (ery)*
HB23172	*WT thrC*::*P*_*yirB(-128*, *+10)*_*-lacZ (ery)*
HB23173	*WT thrC*::*P*_*yirB(-57*, *+46)*_*-lacZ (ery)*
HB23174	*WT thrC*::*P*_*yirB(-57*, *+10)*_*-lacZ (ery)*
HB23175	*ΔcssR thrC*::*P*_*yirB(-128*, *+46)*_*-lacZ (ery)*
HB23176	*ΔcssR thrC*::*P*_*yirB(-128*, *+10)*_*-lacZ (ery)*
HB23177	*ΔcssR thrC*::*P*_*yirB(-57*, *+46)*_*-lacZ (ery)*
HB23178	*ΔcssR thrC*::*P*_*yirB(-57*, *+10)*_*-lacZ (ery)*
HB23179	*ΔyuxN thrC*::*P*_*yirB(-128*, *+46)*_*-lacZ (ery)*
HB23180	*ΔyuxN thrC*::*P*_*yirB(-128*, *+10)*_*-lacZ (ery)*
HB23181	*ΔyuxN thrC*::*P*_*yirB(-57*, *+46)*_*-lacZ (ery)*
HB23182	*ΔyuxN thrC*::*P*_*yirB(-57*, *+10)*_*-lacZ (ery)*
HB23183	*ΔcssR ΔyuxN thrC*::*P*_*yirB(-128*, *+46)*_*-lacZ (ery)*
HB23184	*ΔcssR ΔyuxN thrC*::*P*_*yirB(-128*, *+10)*_*-lacZ (ery)*
HB23185	*ΔcssR ΔyuxN thrC*::*P*_*yirB(-57*, *+46)*_*-lacZ (ery)*
HB23186	*ΔcssR ΔyuxN thrC*::*P*_*yirB(-57*, *+10)*_*-lacZ (ery)*

* All strains are in the HB18501 (168) genetic background and were constructed as part of this study. The *¨Δ¨* symbol is used to indicate a markerless deletion generated using the pDR244 plasmid on a BKE strain from the *Bacillus* Genomic Stock Center (BGSC).

### Strain construction

The knockout mutants were obtained from BGSC (Bacillus Genomic Stock Center), and the erythromycin cassette removed using the plasmid pDR244 [34]. The strains with clean deletions in the *yirB*, *cssR* and *yuxN* genes were constructed by removing the erythromycin resistance cassette from the strains BKE33029, BKE33010, and BKE33030 using the pDR244 plasmid. The BKE knockout mutant strains, the pDR244 plasmid, as well as the transformation method were obtained from the BGSC (*Bacillus* Genomic Stock Center).

The complementation of *yirB* was obtained by PCR amplification of the coding sequence and promoter region with the primers DR242 and DR244 (all primers used are in S1 Table), which were cloned into the EcoR1 and HindIII restriction sites in pDG1662, which integrates into the *amyE* locus (BGSC). Similarly, complementation of *cssR* (primers DR264 and DR259) and *yuxN* (primers DR387 and DR388) was done by integration of corresponding pDG1662 derivatives. The point mutation in the CssR phosphorylation site was obtained using the mutagenic primers DR278 and DR279, which were used for overlap PCR along with the primers DR264 and DR259. The fragment containing the mutation was cloned into pDG1662.

The P_trxB_-lacZ reporter was constructed by PCR amplification of the *trxB* promoter (primers DR112 and DR107) and inserted into pDG1663 (BGSC), which integrates at the *thrC* locus. The vector was amplified using DR104 and DR113, digested with DpnI, and used for Gibson cloning. Similarly, the P_trxA_-*lacZ*, P_yirB_-*lacZ*, and P_yuxN_-*lacZ* transcriptional fusions were constructed by PCR amplification of the promoter using the primers DR404 and DR405, DR242 and DR243, and DR387 and DR408, respectively. The fragments were cloned into the pDG1663 vector. All constructions were verified by PCR and sequencing. For construction of the *yirB* promoter truncations the forward primers DR347, DR348, DR349, DR350 and DR242 with EcoRI restriction site, and the reverse primer DR244 with HindIII restriction site were used to amplify the different fragments. For mutagenesis of the predicted CssRS boxes the mutagenic primers DR305 and DR306, and DR340 and DR341 were used. The primers DR242 and DR244 were used as external primers. The inserts were cloned into pDG1662. All constructions were verified by PCR and sequencing.

For construction of the truncations in Fig 6H, the promoter regions were amplified using the primers: i) DR349 and DR431, ii) DR349 and DR430, iii) DR350 and DR431, and iv) DR350 and DR430. The fragments were then cloned into pDG1663 by restriction cloning. All constructions were verified by PCR and sequencing, and then transformed in HB18501, HB18506, HB23044, and HB23078 to produce the strains HB23171-HB23186.

### Western blot

A total of 5 ml of cells were collected, washed in PBS, and resuspended in 150 μl of disruption Complete EDTA-free Protease Inhibitor Cocktail. The cells were disrupted by sonication, and then centrifuged for 15 min at 13,500 rpm at 4°C. The soluble fraction was collected and quantified using the Bradford Assay. Reducing sample buffer was added to the protein extract, and then 5 μg of protein were loaded in a 4–20% SDS-PAGE. Proteins were transfer onto a PVDF membrane using the TransBlot Turbo Transfer System (Bio-Rad, USA). The membrane was blocked using 5% protein blotting blocker dissolved in TTBS for 1 h. at RT. Then, the primary antibodies were resuspended in 0.5% protein blotting blocker dissolved in TTBS and incubated for 16 h at 4°C. Finally, an anti-rabbit HRP-conjugated secondary antibody was added and incubated for 2 h at RT. The membrane was revealed using the Clarity Western ECL substrate and visualized in a Gel documenter. Protein fractionation was performed as previously described [25]. For quantification of Spx, the intensity of the bands was measured using the Image Lab 5.2.1 software (Bio-Rad, USA)

### β-galactosidase activity

The cells were grown until OD_600_ reached ~0.5. Then, cells were treated or not with different chemicals, and incubated at 37°C with agitation. After specific time points, samples were taken, washed twice in PBS, and finally resuspended in 900 μl of Z buffer (60 mM Na_2_HPO_4_, 40 mM NaH_2_PO_4_, 10 mM KCl, MgSO_4_•7H_2_O) supplemented with 400 μM DTT. Optical density at 600 nm was measured, and then the cells were lysed using 100 μg ml^-1^ lysozyme at 37°C for 30 min. Next, 200 μl of 4 mg ml^-1^ ONPG were added to the lysate, and the reaction was incubated at 28°C until the samples produced a visible yellow color. The reaction was stopped by adding 500 μl of 1.0 M Na_2_CO_3_. The absorbance was then measured at 420 nm and 550 nm, and β-galactosidase activity was determined using the following equation: Miller Units = 1000*[OD_420_-1.75*OD_550_]/(t*v*OD_600_), where *t* is time in minutes and *v* is the volume of culture used in the reaction. It is important to note that the values of β-galactosidase activity after treatment with cell wall active antibiotics might underestimate the effect of the drug on gene expression. This result was previously noted using another stable protein reporter (i.e. GFP) and is due to partial lysis elicited by antibiotic treatment.

### Northern blot

RNA was isolated using the hot phenol-chloroform method as previously described [4]. RNA concentration and purity were determined using spectrophotometry, while RNA integrity was checked using denaturing agarose gels. Northern blot was performed on nylon membranes using radiolabeled RNA probes as previously described [4]. The *yirB* RNA probe was obtained using the primers P45 and DR282; the *htrB* probe was generated using the primers P47 and DR283; and the *spx* probe was obtained using the primers DR319 and DR320.

### RT-qPCR

The RT-qPCR was performed as previously reported [4]. The primers used for *spx* were P11 and P12, for *sigM* were P31 and P32, for *trxB* were P13 and P14, for *yjbH* were P17 and P18, for *gyrA* were P33 and P34, for *yirB* were P45 and P46, for *htrB* were P47 and P48, and for *murAA* were P3 and P4.

### Protein chase experiments

In order to determine the stability of Spx in WT and Δ*yirB*, cells were grown on 50 ml of LB broth up to OD_600_ = 0.5. Then vancomycin was added to a final concentration of 1 μg ml^-1^ to induce the stress response, cells were incubated for 10 min at 37°C with shaking, and then pre-warmed chloramphenicol [100 μg ml^-1^, final concentration] was added to stop protein synthesis. Samples (1.5 ml) were taken after 0, 1, 2, 3, and 5 min, and proteolysis was stopped by mixing the cells with 150 μl of pre-chilled 100% trichloroacetic acid (TCA). Cell suspensions were centrifuged at 13,500 rpm for 10 min at 4°C, and the pellet was washed twice with ice-cold acetone in order to remove all TCA. The cell pellets were air dried for 10 min, resuspended in 130 μL of solubilization buffer (1% SDS, 1 mM EDTA, 100 mM Tris-HCl, pH 8.0), and sonicated. A volume of 5 μl of the protein sample was load in a 4–20% SDS-PAGE gel, and western blot was carried out as previously described.

### 5’ RACE

Cells were grown up to OD_600_ = 0.5 and treated with 1 μg ml^-1^ vancomycin. After 10 min of incubation, 5 ml of sample were collected, and RNA was isolated using the RNeasy Kit (Qiagen) following manufacturer’s instructions. The RNA was treated with Turbo DNase, and then purified by phenol-chloroform extraction. The RNA was quantified using a Nanodrop, its purity assessed by the 260/280 ratio, and integrity monitored by agarose gel electrophoresis. A total of 1 μg of RNA was reverse transcribed using the P46 primer and the Reverse Transcription Reagents (Thermo Fisher Scientific, US) following manufacturer’s instructions. The cDNA was column purified and then treated with the terminal transferase enzyme using CTP to add a homopolymeric cytosine tail at the 3’ end. Then, the cDNA was PCR amplified using the abridged anchor primer (AAP) and DR288 primers by using a touchdown PCR followed by a conventional PCR. The PCR product was verified by electrophoresis and sequenced using the DR289 primer.

## Supporting information

S1 Table Primer sequences. (PDF)

S1 Fig YirB also affects *trxB* induction in cells with conditional expression of *spx*. The induction of the Spx-dependent gene *trxB* was monitored 0 min. and 30 min. after treatment using a P_*trxB*_-*lacZ* transcriptional fusion integrated at the *thrC* locus. (A) The *spx* gene was constitutively expressed by addition of various concentrations of IPTG (i.e. 20 μM, 60 μM, and 100 μM), and then the cells were treated or not with 1 μg ml^-1^vancomycin. (B) The expression of *spx* was artificially induced. For this, cells were grown in the presence of 20 μM IPTG, and induction of the gene was achieved by using IPTG to reach 20 μM, 60 μM and 100 μM IPTG. Cells were treated or not with 1 μg ml^-1^ vancomycin. Error bars represent SEM of at least three independent replicates. One, two, and three asterisks indicate significant differences with P < 0.05, P < 0.01 and P < 0.001 respectively, as estimated using one-way ANOVA and the Tukey’s HSD test. NS indicates no significant differences.
(PDF)

S2 Fig YjbH aggregation in response to vancomycin and diamide treatment. YjbH-HA was studied in the soluble and insoluble protein fractions after treatment with 1 μg ml^-1^ vancomycin and 500 μM diamide. As observed, only diamide led to a significant accumulation of YjbH in the insoluble fraction.
(PDF)

S3 Fig CssR binding sites. (A) The known CssR boxes in the *Bacillus subtilis* 168 genome were aligned to determine the CssR box consensus sequence. (B) A DNA logo was created for the consensus CssR box.
(PDF)

S4 Fig Palindromic sequences in the *yirB* and *yuxN* promoters. (A) Promoter region of the *yirB* and *yuxN* promoters in different *Bacillus* species. In descending order, *Bacillus amyloliquefaciens* DSM7 [NC_014551.1], *Bacillus subtilis* strain 168 [NC_000964.3], *Bacillus gibsonii* strain FJAT-10019 [CP17070.1], *Bacillus atrophaeus* strain [SRCM101359], and *Bacillus licheniformis* strain [SRCM101441]. The mapped *yirB* promoter and the predicted *yuxN* promoter are also displayed, as well as the palindrome sequences found on both promoters. (B) DNA logos were created for the *yirB* and *yuxN* palindromes.
(PDF)

S5 Fig Effect of truncations, point mutations, and gene deletions on induction of the Spx-controlled gene *trxB*. (A) Analysis of basal *trxB* activity in cells expressing the different *yirB* promoter truncations. Statistical analysis was performed in pairs using the T-test. (B) Analysis of basal *trxB* activity in cells expressing the wild-type *yirB* promoter vs. the mutant *yirB* promoters harboring point mutations in the CssR boxes. Statistical analysis was performed using the Dunnett Test, comparing P_*yirB*(-538)_ against promoters of the same length but including mutations in the CssR predicted boxes. (C) Effect of the deletions of CssR, YuxN, and CssR & YuxN on basal expression levels of *trxB*. Statistical analysis was performed using the Dunnett Test comparing the mutant strains against WT. Error bars represent SEM of at least three independent replicates. One, two, and three asterisks indicate significant differences with P < 0.05, P < 0.01 and P < 0.001 respectively. NS indicates no significant differences.
(PDF)

S6 Fig Εffect of deletions of major transcription regulators involved in the cell wall stress response on activation of the CssRS regulon. Induction of the CssR regulon in response to vancomycin treatment in cells lacking SigM, SigW, or LiaR.
(PDF)

## References

[1] Browning DF, Busby SJW. Local and global regulation of transcription initiation in bacteria. Nat Rev Microbiol. 2016;14: 638–650. 10.1038/nrmicro.2016.103 27498839

[2] Helmann JD. *Bacillus subtilis* extracytoplasmic function (ECF) sigma factors and defense of the cell envelope. Curr Opin Microbiol. 2016;30: 122–132. 10.1016/j.mib.2016.02.002 26901131 PMC4821709

[3] Jordan S, Hutchings MI, Mascher T. Cell envelope stress response in Gram-positive bacteria. FEMS Microbiol Rev. 2008;32: 107–146. 10.1111/j.1574-6976.2007.00091.x 18173394

[4] Rojas Tapias DF, Helmann JD. Induction of the Spx regulon by cell wall stress reveals novel regulatory mechanisms in *Bacillus subtilis*. Mol Microbiol. 2018;107: 659–674. 10.1111/mmi.13906 29271514 PMC5820111

[5] Kirstein J, MoliEre N, Dougan DA, Turgay K. Adapting the machine: adaptor proteins for Hsp100/Clp and AAA^+^ proteases. Nat Rev Microbiol. 2009;7: 589–599. 10.1038/nrmicro2185 19609260

[6] Bougdour A, Wickner S, Gottesman S. Modulating RssB activity: IraP, a novel regulator of sigma(S) stability in *Escherichia coli*. Genes Dev. 2006;20: 884–897. 10.1101/gad.1400306 16600914 PMC1472289

[7] Bougdour A, Cunning C, Baptiste PJ, Elliott T, Gottesman S. Multiple pathways for regulation of σ^S^ (RpoS) stability in *Escherichia coli* via the action of multiple anti-adaptors. Mol Microbiol. 2008;68: 298–313. 10.1111/j.1365-2958.2008.06146.x 18383615

[8] Battesti A, Gottesman S. Roles of adaptor proteins in regulation of bacterial proteolysis. Curr Opin Microbiol. Elsevier Ltd; 2013;16: 140–147. 10.1016/j.mib.2013.01.002 23375660 PMC3646950

[9] Nakano S, Küster-Schöck E, Grossman AD, Zuber P. Spx-dependent global transcriptional control is induced by thiol-specific oxidative stress in *Bacillus subtilis*. Proc Natl Acad Sci USA. 2003;100: 13603–13608. 10.1073/pnas.2235180100 14597697 PMC263860

[10] Pamp SJ, Frees D, Engelmann S, Hecker M, Ingmer H. Spx Is a Global Effector Impacting Stress Tolerance and Biofilm Formation in *Staphylococcus aureus*. J Bacteriol. 2006;188: 4861–4870. 10.1128/JB.00194-06 16788195 PMC1483011

[11] Veiga P, Bulbarela-Sampieri C, Furlan S, Maisons A, Chapot-Chartier M-P, Erkelenz M, et al SpxB regulates O-acetylation-dependent resistance of *Lactococcus lactis* peptidoglycan to hydrolysis. J Biol Chem. 2007;282: 19342–19354. 10.1074/jbc.M611308200 17485463

[12] Turlan C, Prudhomme M, Fichant G, Martin B, Gutierrez C. SpxA1, a novel transcriptional regulator involved in X-state (competence) development in *Streptococcus pneumoniae*. Mol Microbiol. 2009;73: 492–506. 10.1111/j.1365-2958.2009.06789.x 19627499

[13] Runde S, MoliEre N, Heinz A, Maisonneuve E, Janczikowski A, Elsholz AKW, et al The role of thiol oxidative stress response in heat-induced protein aggregate formation during thermotolerance in *Bacillus subtilis*. Mol Microbiol. 2014;91: 1036–1052. 10.1111/mmi.12521 24417481

[14] Rochat T, Nicolas P, Delumeau O, Rabatinova A, Korelusova J, Leduc A, et al Genome-wide identification of genes directly regulated by the pleiotropic transcription factor Spx in *Bacillus subtilis*. Nucleic Acids Res. 2012;40: 9571–9583. 10.1093/nar/gks755 22904090 PMC3479203

[15] Gaballa A, Antelmann H, Hamilton CJ, Helmann JD. Regulation of *Bacillus subtilis* bacillithiol biosynthesis operons by Spx. Microbiology. 2013;159: 2025–2035. 10.1099/mic.0.070482-0 23894131

[16] Eiamphungporn W, Helmann JD. The *Bacillus subtilis* σ^M^ regulon and its contribution to cell envelope stress responses. Mol Microbiol. 2008;67: 830–848. 10.1111/j.1365-2958.2007.06090.x 18179421 PMC3025603

[17] Antelmann H, Scharf C, Hecker M, Hecker M. Phosphate Starvation-Inducible Proteins of *Bacillus subtilis*: Proteomics and Transcriptional Analysis. J Bacteriol. American Society for Microbiology; 2000;182: 4478–4490. 10.1128/JB.182.16.4478–4490.2000 PMC94619 10913081

[18] Leelakriangsak M, Zuber P. Transcription from the P_3_ promoter of the *Bacillus subtilis spx* gene is induced in response to disulfide stress. J Bacteriol. 2007;189: 1727–1735. 10.1128/JB.01519-06 17158663 PMC1855742

[19] Leelakriangsak M, Kobayashi K, Zuber P. Dual negative control of *spx* transcription initiation from the P_3_ promoter by repressors PerR and YodB in *Bacillus subtilis*. J Bacteriol. 2007;189: 1736–1744. 10.1128/JB.01520-06 17158660 PMC1855716

[20] Nakano S, Erwin KN, Ralle M, Zuber P. Redox-sensitive transcriptional control by a thiol/disulphide switch in the global regulator, Spx. Mol Microbiol. 2005;55: 498–510. 10.1111/j.1365-2958.2004.04395.x 15659166

[21] Nakano S, Zheng G, Nakano MM, Zuber P. Multiple pathways of Spx (YjbD) proteolysis in *Bacillus subtilis*. J Bacteriol. 2002;184: 3664–3670. 10.1128/JB.184.13.3664-3670.2002 12057962 PMC135134

[22] Garg SK, Kommineni S, Henslee L, Zhang Y, Zuber P. The YjbH protein of *Bacillus subtilis* enhances ClpXP-catalyzed proteolysis of Spx. J Bacteriol. 2009;191: 1268–1277. 10.1128/JB.01289-08 19074380 PMC2632004

[23] Larsson JT, Rogstam A, Wachenfeldt von C. YjbH is a novel negative effector of the disulphide stress regulator, Spx, in *Bacillus subtilis*. Mol Microbiol. 2007;66: 669–684. 10.1111/j.1365-2958.2007.05949.x 17908206

[24] Chan CM, Hahn E, Zuber P. Adaptor bypass mutations of *Bacillus subtilis spx* suggest a mechanism for YjbH-enhanced proteolysis of the regulator Spx by ClpXP. Mol Microbiol. 2014;93: 426–438. 10.1111/mmi.12671 24942655 PMC4128009

[25] Engman J, Wachenfeldt von C. Regulated protein aggregation: a mechanism to control the activity of the ClpXP adaptor protein YjbH. Mol Microbiol. 2015;95: 51–63. 10.1111/mmi.12842 25353645

[26] Kommineni S, Garg SK, Chan CM, Zuber P. YjbH-enhanced proteolysis of Spx by ClpXP in *Bacillus subtilis* is inhibited by the small protein YirB (YuzO). J Bacteriol. 2011;193: 2133–2140. 10.1128/JB.01350-10 21378193 PMC3133067

[27] Hyyryläinen HL, Bolhuis A, Darmon E, Muukkonen L, Koski P, Vitikainen M, et al A novel two-component regulatory system in *Bacillus subtilis* for the survival of severe secretion stress. Mol Microbiol. 2001;41: 1159–1172. 11555295 10.1046/j.1365-2958.2001.02576.x

[28] Wecke T, Bauer T, Harth H, Mäder U, Mascher T. The rhamnolipid stress response of *Bacillus subtilis*. FEMS Microbiol Lett. 2011;323: 113–123. 10.1111/j.1574-6968.2011.02367.x 22092710

[29] Cao M, Wang T, Ye R, Helmann JD. Antibiotics that inhibit cell wall biosynthesis induce expression of the *Bacillus subtilis* σ^W^ and σ^M^ regulons. Mol Microbiol. 2002;45: 1267–1276. 10.1046/j.1365-2958.2002.03050.x 12207695

[30] Nicolas P, Mäder U, Dervyn E, Rochat T, Leduc A, Pigeonneau N, et al Condition-dependent transcriptome reveals high-level regulatory architecture in *Bacillus subtilis*. Science (New York, NY). 2012;335: 1103–1106. 10.1126/science.1206848 22383849

[31] Darmon E, Noone D, Masson A, Bron S, Kuipers OP, Devine KM, et al A Novel Class of Heat and Secretion Stress-Responsive Genes Is Controlled by the Autoregulated CssRS Two-Component System of *Bacillus subtilis*. J Bacteriol. 2002;184: 5661–5671. 10.1128/JB.184.20.5661-5671.2002 12270824 PMC139597

[32] Zhang Y, Zuber P. Requirement of the zinc-binding domain of ClpX for Spx proteolysis in *Bacillus subtilis* and effects of disulfide stress on ClpXP activity. J Bacteriol. 2007;189: 7669–7680. 10.1128/JB.00745-07 17827297 PMC2168722

[33] Noone D, Botella E, Butler C, Hansen A, Jende I, Devine KM. Signal Perception by the Secretion Stress-Responsive CssRS Two-Component System in *Bacillus subtilis*. J Bacteriol. 2012;194: 1800–1814. 10.1128/JB.05767-11 22307758 PMC3302470

[34] Koo B-M, Kritikos G, Farelli JD, Todor H, Tong K, Kimsey H, et al Construction and Analysis of Two Genome-Scale Deletion Libraries for *Bacillus subtilis*. Cell Syst. 2017;4: 291–305.e7. 10.1016/j.cels.2016.12.013 28189581 PMC5400513

