# Supplementary material for: Stabilization of Bacillus subtilis Spx under cell wall stress requires the anti-adaptor protein YirB
Source: PLoS Genet. 2018 Jul 12;14(7):e1007531. doi: 10.1371/journal.pgen.1007531 (PMC6057675; doi:10.1371/journal.pgen.1007531)
Supplement: S1 Table — (PDF) [file pgen.1007531.s001.pdf]

Table S1. Primer sequences

Primer	*Sequence*
DR104	GCTTTTATATAGGGAAAAGGTGGTG
DR107	CCACCTTTTCCCTATATAAAAGCGGAATCTCCGCCGCCAACAAC
DR112	GGATCCCCAGCTTGTTGATACACGGACACGTAGGAATCTACTTAGG
DR113	GTGTATCAACAAGCTGGGGATCC
DR242	ATCAGAATTCCGCTTTTTTTCCATAGATGTTCAG
DR243	ATCAGGATCCCTCATTCTAGATTCACCCTTTCG
DR244	ATCGAAGCTTGACAAAACGGGTGTAACATACC
DR259	ATCGAAGCTTGGATCCTCATGATGACATCATCCTGTAGC
DR264	ATCGAGAATTCCTTACACTCCTTTAACGGTTATTC
DR278	GGATTCTCGCTATCATGCTGCCG
DR279	GCAGCATGATAGCGAGAATCCATAGGTGGGGAGACGGTGTC
DR282	ATCGATAATACGACTCACTATAGGTGTGAAAGAATAGCCGTTAAAATC
DR283	ATCGATAATACGACTCACTATAGGGAGAATATCCGCACTGCTTTCCAC
DR288	GCTGTTTGCATTGATGAACC
DR289	CATTTTTTCTCTGACAAGGTCTG
DR305	CCGTCAGAAATAAAATTAAACTTATTGTAACTTG
DR306	AGTTTAATTTTATTTCTGACGGTGTGACTATATGGTC
DR319	TGTACCGCTTGATCAACGAG
DR320	ATCGATAATACGACTCACTATAGGGAGAGCCAAACGCTGTGCTTCTC
DR340	GATATGACTTTTTAGATATTCCGTCTGTTATAAAATTAACCTTATTG
DR341	AATATCTAAAAAGTCATATCCTAGCAGGCCTCC
DR347	ATCAGAATTCGAATGGTCAAAGCTCAAGCG
DR348	ATCAGAATTCTTATTGTAACTTGTTTTTTTATC
DR349	ATCAGAATTCTCTGTTATAAAATTAACCTTATTGTAAC
DR350	ATCAGAATTCCCTTCAATGGTTCCACCCTTTC
DR387	ATCAGAATTCTTCACCCTTTCGCTTGAGCTTTG
DR388	ATCAGGATCCCTGACAGAGGAGCAATTTAACG
DR404	ATCAAAGCTTGTCAGTCGTCTTACTTACGCTG
DR405	ATCAGGATCCGATCAGTTGCTTTTACGATAGC
DR408	ATCAGGATCCCAGTTTTTCTTTTTCCTTCAATGG
DR430	ATCAGGATCCTCTCAATTTATAATATAAATAATACATG
DR431	ATCAGGATCCCCTTTCGCTTGAGCTTTGACC
P3	GCTCCTACACTTGGGAAGTCCAG
P4	GCAAGAGTTGCACTTCCGGG
P11	CCAAGCTGTACTTCATGCAGAAAGGC
P12	GATCAAGCGGTACAAATCTTGCAGC

P13	GCGTGCCTGGCGAAAAAGAATTGG
P14	GAGCACGGAGTTTATCACGTCTGTG
P17	GATGAAAACGTGCTTTTGGAGATTGCTG
P18	CTTTAAGCCCTTCATCCTCATGCTGC
P31	TGATCACAGCAAAGTAAAGCCCTGGC
P32	CAGGACTTTGAACAGCATTTTGAAAGAGG
P33	GCAACAAACATTCCTCCGCACCAG
P34	CTTTTGCCCGGATCGTGATAGAGC
P45	GAATTGGACGAAATGATCAGC
P46	TGTGAAAGAATAGCCGTTAAAATC
P47	TTTAGGCGTGCAGATGATTG
P48	ATATCCGCACTGCTTTCCAC
