# Supplementary material for: Stabilization of Bacillus subtilis Spx under cell wall stress requires the anti-adaptor protein YirB
Source: PLoS Genet. 2018 Jul 12;14(7):e1007531. doi: 10.1371/journal.pgen.1007531 (PMC6057675; doi:10.1371/journal.pgen.1007531)
Supplement: S1 Fig — The induction of the Spx-dependent gene trxB was monitored 0 min. and 30 min. after treatment using a PtrxB-lacZ transcriptional fusion integrated at the thrC locus. (A) The spx gene was constitutively expressed by addition of various concentrations of IPTG (i.e. 20 μM, 60 μM, and 100 μM), and then the cells were treated or not with 1 μg ml-1vancomycin. (B) The expression of spx was artificially induced. For this, cells were grown in the presence of 20 μM IPTG, and induction of the gene was achieved by using IPTG to reach 20 μM, 60 μM and 100 μM IPTG. Cells were treated or not with 1 μg ml-1 vancomycin. Error bars represent SEM of at least three independent replicates. One, two, and three asterisks indicate significant differences with P < 0.05, P < 0.01 and P < 0.001 respectively, as estimated using one-way ANOVA and the Tukey’s HSD test. NS indicates no significant differences. (PDF) [file pgen.1007531.s002.pdf]

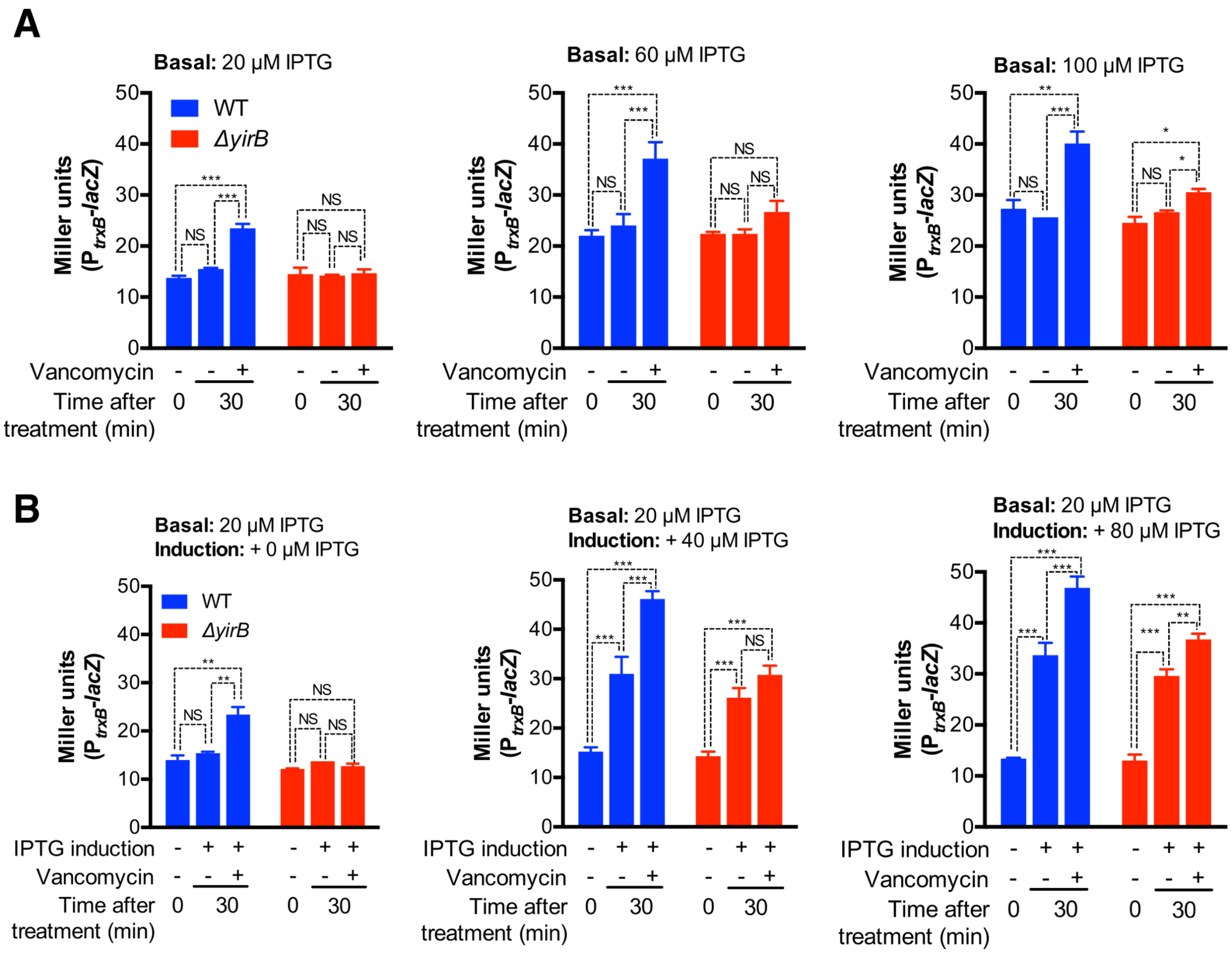

Fig S1 YirB also affects *trxB* induction in cells with conditional expression of *spx*. The induction of the Spx-dependent gene *trxB* was monitored 0 min. and 30 min. after treatment using a P_{trxB}-*lacZ* transcriptional fusion integrated at the *thrC* locus. A) The *spx* gene was constitutively expressed by addition of various concentrations of IPTG (i.e. 20 μM, 60 μM, and 100 μM), and then the cells were treated or not with 1 μg ml^{-1}vancomycin. B) The expression of *spx* was artificially induced. For this, cells were grown in the presence of 20 μM IPTG, and induction of the gene was achieved by using IPTG to reach 20 μM, 60 μM and 100 μM IPTG. Cells were treated or not with 1 μg ml^{-1} vancomycin. Error bars represent SEM of at least three independent replicates. One, two, and three asterisks indicate significant differences with $P < 0.05$, $P < 0.01$ and $P < 0.001$ respectively, as estimated using one-way ANOVA and the Tukey's HSD test. NS indicates no significant differences.
