# Supplementary material for: Stabilization of Bacillus subtilis Spx under cell wall stress requires the anti-adaptor protein YirB
Source: PLoS Genet. 2018 Jul 12;14(7):e1007531. doi: 10.1371/journal.pgen.1007531 (PMC6057675; doi:10.1371/journal.pgen.1007531)
Supplement: S2 Fig — YjbH-HA was studied in the soluble and insoluble protein fractions after treatment with 1 μg ml-1 vancomycin and 500 μM diamide. As observed, only diamide led to a significant accumulation of YjbH in the insoluble fraction. (PDF) [file pgen.1007531.s003.pdf]

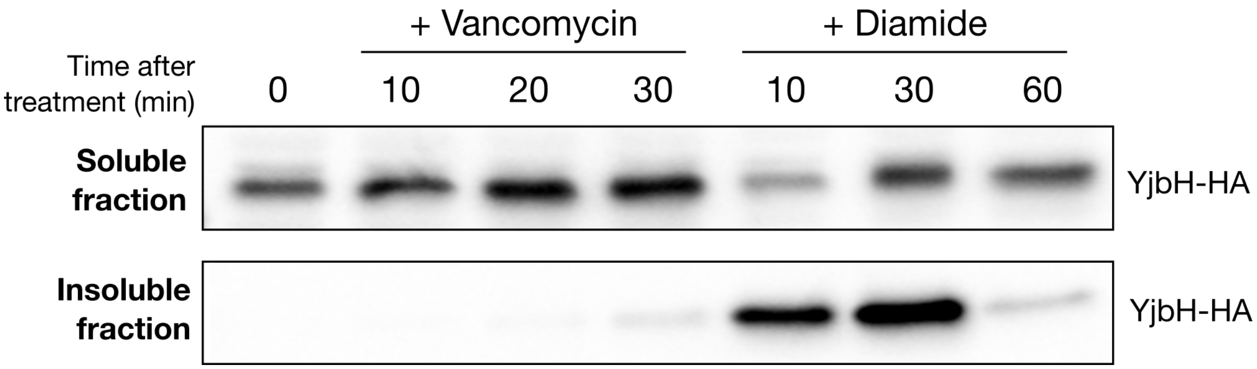

Fig S2 YjbH aggregation in response to vancomycin and diamide treatment.

YjbH-HA was studied in the soluble and insoluble protein fractions after treatment with 1 μg ml^{-1} vancomycin and 500 μM diamide. As observed, only diamide led to a significant accumulation of YjbH-HA in the insoluble fraction.
