# Supplementary material for: Stabilization of Bacillus subtilis Spx under cell wall stress requires the anti-adaptor protein YirB
Source: PLoS Genet. 2018 Jul 12;14(7):e1007531. doi: 10.1371/journal.pgen.1007531 (PMC6057675; doi:10.1371/journal.pgen.1007531)
Supplement: S3 Fig — (A) The known CssR boxes in the Bacillus subtilis 168 genome were aligned to determine the CssR box consensus sequence. (B) A DNA logo was created for the consensus CssR box. (PDF) [file pgen.1007531.s004.pdf]

A

```
CLUSTAL multiple sequence alignment by MUSCLE (3.8)


htrB            TTTTCATTTTTTATCCCA
htrA2           TTTTCATTTTTTATCCCA
cssR            TTTTCATAATT-TCACA
htrA            TTTTCACAATT-TCCCA
                ******   ** ** **
```

B

Consensus CssR binding site based on the alignment above. The CssR response regulator is predicted to bind as a dimer on sequences repeated in tandem.

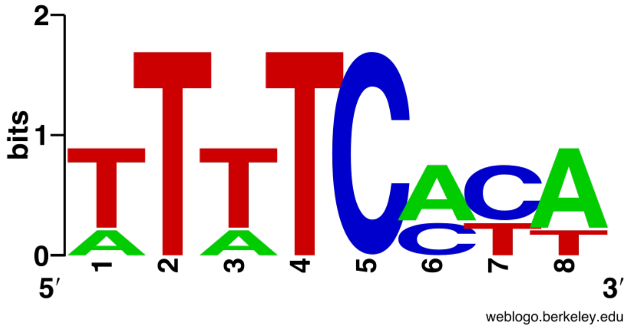

Fig S3 CssR binding sites. A) The known CssR boxes in the *Bacillus subtilis* 168 genome were aligned to determine the CssR box consensus sequence. B) A DNA logo was created for the consensus CssR box.
