# Supplementary material for: Stabilization of Bacillus subtilis Spx under cell wall stress requires the anti-adaptor protein YirB
Source: PLoS Genet. 2018 Jul 12;14(7):e1007531. doi: 10.1371/journal.pgen.1007531 (PMC6057675; doi:10.1371/journal.pgen.1007531)
Supplement: S4 Fig — (A) Promoter region of the yirB and yuxN promoters in different Bacillus species. In descending order, Bacillus amyloliquefaciens DSM7 [NC_014551.1], Bacillus subtilis strain 168 [NC_000964.3], Bacillus gibsonii strain FJAT-10019 [CP17070.1], Bacillus atrophaeus strain [SRCM101359], and Bacillus licheniformis strain [SRCM101441]. The mapped yirB promoter and the predicted yuxN promoter are also displayed, as well as the palindrome sequences found on both promoters. (B) DNA logos were created for the yirB and yuxN palindromes. (PDF) [file pgen.1007531.s005.pdf]

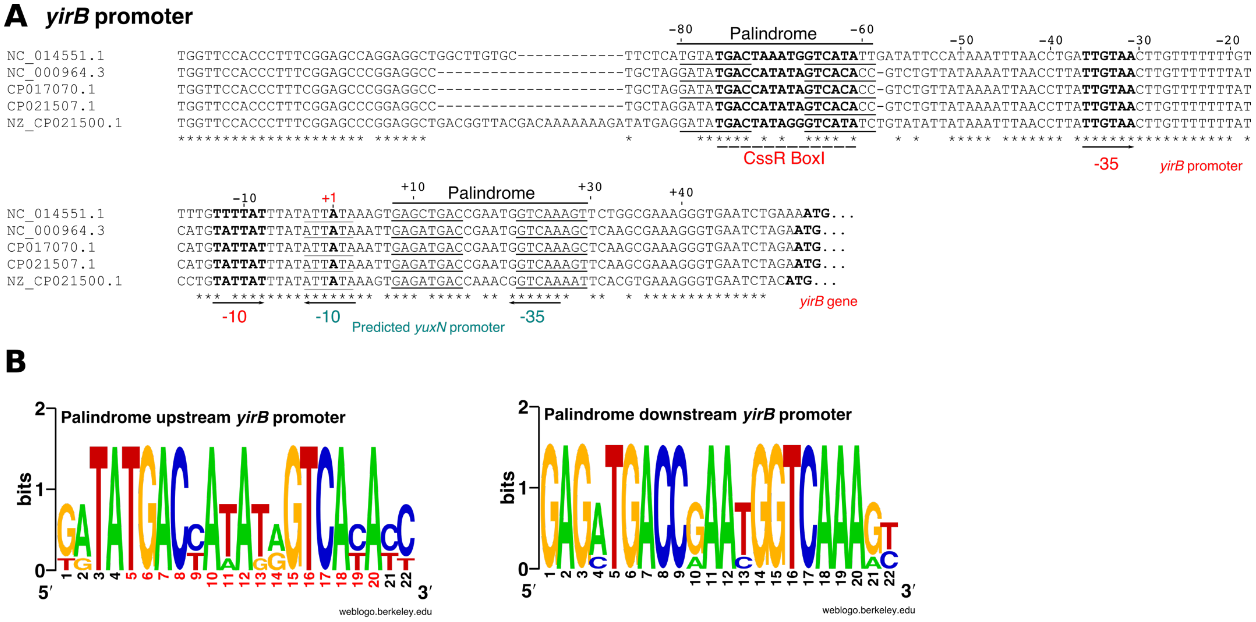

Fig S4 Palindromic sequences in the *yirB* and *yuxN* promoters. A) Promoter region of the *yirB* and *yuxN* promoters in different *Bacillus* species. In descending order, *Bacillus amyloliquefaciens* DSM7 [NC_014551.1], *Bacillus subtilis* strain 168 [NC_000964.3], *Bacillus gibsonii* strain FJAT-10019 [CP17070.1], *Bacillus atrophaeus* strain [SRCM101359], and *Bacillus licheniformis* strain [SRCM101441]. The mapped *yirB* promoter and the predicted *yuxN* promoter are also displayed, as well as the palindrome sequences found on both promoters. B) DNA logos were created for the *yirB* and *yuxN* palindromes, as observed they display remarkable similarity.
