# Supplementary material for: Stabilization of Bacillus subtilis Spx under cell wall stress requires the anti-adaptor protein YirB
Source: PLoS Genet. 2018 Jul 12;14(7):e1007531. doi: 10.1371/journal.pgen.1007531 (PMC6057675; doi:10.1371/journal.pgen.1007531)
Supplement: S5 Fig — (A) Analysis of basal trxB activity in cells expressing the different yirB promoter truncations. Statistical analysis was performed in pairs using the T-test. (B) Analysis of basal trxB activity in cells expressing the wild-type yirB promoter vs. the mutant yirB promoters harboring point mutations in the CssR boxes. Statistical analysis was performed using the Dunnett Test, comparing PyirB(-538) against promoters of the same length but including mutations in the CssR predicted boxes. (C) Effect of the deletions of CssR, YuxN, and CssR & YuxN on basal expression levels of trxB. Statistical analysis was performed using the Dunnett Test comparing the mutant strains against WT. Error bars represent SEM of at least three independent replicates. One, two, and three asterisks indicate significant differences with P < 0.05, P < 0.01 and P < 0.001 respectively. NS indicates no significant differences. (PDF) [file pgen.1007531.s006.pdf]

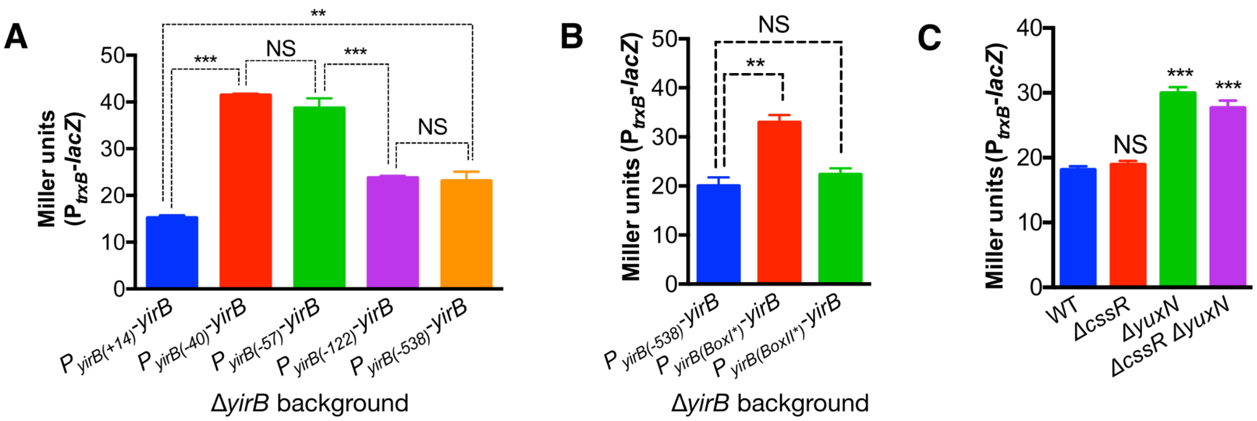

Fig S5 Effect of truncations, point mutations, and gene deletions on induction of the Spx-controlled gene *trxB*. A) Analysis of basal *trxB* activity in cells expressing the different *yirB* promoter truncations. Statistical analysis was performed in pairs using the T-test. B) Analysis of basal *trxB* activity in cells expressing the wild-type *yirB* promoter vs. the mutant *yirB* promoters harboring point mutations in the CssR BoxI boxes. Statistical analysis was performed using the Dunnett Test, comparing $P_{yirB(-538)}$ against promoters of the same length but including mutations in the CssR predicted boxes. C) Effect of the deletions of CssR, YuxN, and CssR & YuxN on basal expression levels of *trxB*. Statistical analysis was performed using the Dunnett Test comparing the mutant strains against WT. Error bars represent SEM of at least three independent replicates. One, two, and three asterisks indicate significant differences with P < 0.05, P < 0.01 and P < 0.001 respectively. NS indicates no significant differences.
