# Supplementary material for: Stabilization of Bacillus subtilis Spx under cell wall stress requires the anti-adaptor protein YirB
Source: PLoS Genet. 2018 Jul 12;14(7):e1007531. doi: 10.1371/journal.pgen.1007531 (PMC6057675; doi:10.1371/journal.pgen.1007531)
Supplement: S6 Fig — Induction of the CssR regulon in response to vancomycin treatment in cells lacking SigM, SigW, or LiaR. (PDF) [file pgen.1007531.s007.pdf]

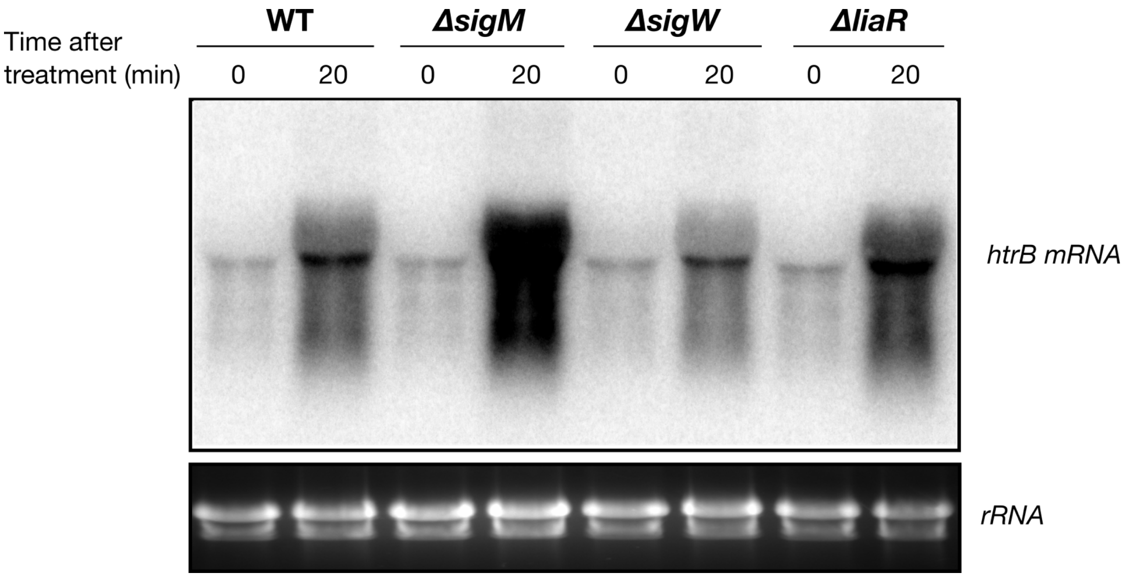

Fig S6 Effect of deletions of major transcription regulators involved in the cell wall stress response on activation of the CssRS regulon. Induction of the CssR regulon in response to vancomycin treatment in cells lacking SigM, SigW, or LiaR.
